# Comparing Two Models of Transition from Inpatient Rehabilitation Following Traumatic Brain Injury: A Pragmatic Comparative Effectiveness Trial

**DOI:** 10.1177/08977151251374298

**Published:** 2025-09-17

**Authors:** Jeanne M. Hoffman, Taylor Obata, Marcia A. Ciol, Andrew Humbert, Jennifer Bogner, John D. Corrigan, Kristen Dams-O’Connor, Simon Driver, Rosemary Dubiel, Flora M. Hammond, Tessa Hart, Maria Kajankova, Megan Moore, Thomas K. Watanabe, John Whyte, Jesse R. Fann

**Affiliations:** 1 Department of Rehabilitation Medicine, University of Washington School of Medicine, Seattle, Washington, USA.; 2 Department of Physical Medicine and Rehabilitation, College of Medicine, The Ohio State University, Columbus, Ohio, USA.; 3 Department of Rehabilitation and Human Performance, Icahn School of Medicine at Mount Sinai, New York, New York, USA.; 4 Department of Neurology, Icahn School of Medicine at Mount Sinai, New York, New York, USA.; 5 Department of Physical Medicine and Rehabilitation, Baylor Scott and White Research Institute, Dallas, Texas, USA.; 6 Department of Physical Medicine and Rehabilitation, Indiana University School of Medicine, Indianapolis, Indiana, USA.; 7 Moss Rehabilitation Research Institute, Elkins Park, Pennsylvania, USA.; 8 School of Social Work and Harborview Injury Prevention and Research Center, University of Washington, Seattle, Washington, USA.; 9 MossRehab at Elkins Park/Einstein Healthcare Network, Elkins Park, Pennsylvania, USA.; 10 Department of Psychiatry and Behavioral Sciences, University of Washington School of Medicine, Seattle, Washington, USA.

**Keywords:** brain injury, case management, inpatient rehabilitation, traumatic brain injury, transition of care, quality of life

## Abstract

Moderate to severe traumatic brain injury (msTBI) results in physical, cognitive, behavioral, and psychosocial difficulties. Those who receive inpatient rehabilitation following a msTBI need assistance after discharge. Patients and their families often struggle to find information, manage symptoms, and identify and access relevant services. Inadequate transition services from hospital-based care to the community can perpetuate and amplify the consequences of msTBI. There is a critical need for enhanced transitional care following hospital discharge. The goal of the current study was to compare two existing models for supporting the transition in the United States: 1. The Commission on Accreditation of Rehabilitation Facilities (CARF) model focused on transition planning prior to discharge (denominated Rehabilitation Discharge Plan [RDP]) and 2. The Veterans Health Administration model which provides a more intensive approach, extending beyond discharge, to enhance transitional care services (denominated Rehabilitation Transition Plan [RTP]). A six-center, 1:1 randomized pragmatic clinical trial with masked outcome assessment was conducted to compare the effectiveness of these two approaches. All participants received the RDP, including: 1. Patient and family education; 2. Written discharge instructions reviewed with the patient and family prior to discharge; and 3. A brief phone call from an inpatient care provider post discharge to identify any immediate problems. Those randomized to the RTP intervention also received up to 12 scheduled contacts during the 6 months following discharge from a trained care manager to assess needs, provide education, and resource facilitation. The primary outcomes were societal participation (participation assessment with recombined toolsobjective) and quality of life (quality of life after brain injury scale) at 6 months post discharge. We hypothesized that patients randomized to RTP would report better participation and health-related quality of life (HRQoL) at the end of intervention and at 1-year post discharge compared with patients randomized to RDP. Second, we hypothesized that patients randomized to RTP would experience a steeper trajectory of improvement in participation and HRQoL over 12 months compared to patients randomized to RDP. A total of 925 patients were randomized. The results showed no significant differences between the two interventions on societal participation or HRQoL. Likewise, analysis of trajectory of outcomes did not show treatment group differences, and most patient participants had minimal change across all time points. Preinjury limitations, Medicaid insurance, and lower function contributed to worse outcomes but there was evidence for an interaction with the intervention for clinical sites and whether participants had an enrolled caregiver, which differed by group (increased participation with no enrolled caregiver in RTP, and better HRQoL with a caregiver for RDP). A key limitation of the study was the length of the intervention, with our patient, family, and professional partners reporting that 6 months might be insufficient to address the many needs that arise after msTBI.

## Introduction

Approximately 215,000 traumatic brain injury (TBI)-related hospitalizations occurred in the United States in 2020^1^ and, of those, nearly half resulted in long-term disability.^2^ Moderate to severe TBI (msTBI) commonly results in a complex mix of physical, cognitive, behavioral, and psychosocial difficulties; the cognitive sequelae in particular may limit one’s ability to adjust to functional changes and manage long-term health and rehabilitation needs.^2^ msTBI also frequently evolves into a chronic disease process that can include reduced life span and the development and/or exacerbation of comorbid conditions,^3–6^ requiring ongoing treatment. Structural barriers to recovery also contribute to differences in outcome and include drivers of health inequity, such as racism, language discrimination, economic insecurity, and location (e.g., rurality).^7–15^ Brain injury outcome is affected by the type, location in the brain where the injury occurs and severity of injury, and is mitigated by modifiable factors such as: 1. access to specialized trauma care; 2. intensity, duration, timing, setting, and scope of rehabilitation; and 3. the availability of and access to follow-up services.^16–25^ However, despite the extent to the need for specialized care, only about 13–25% of those with msTBI receive inpatient rehabilitation facility (IRF) level care.^26^

For those who receive treatment from an IRF, many report unmet needs^27^ as they struggle to find information about their TBI, manage their symptoms, and identify and access relevant services post-discharge.^28,29^ Even among those fortunate enough to receive IRF care, inadequate transition services from hospital-based care to the community can perpetuate and over time amplify the consequences of msTBI, which can include ruptured interpersonal relationships, high rates of divorce and separation, caregiver distress and burden, and mood disorders.^30^ This underscores the critical need for enhanced discharge planning and transitional care services following hospital discharge^31–35^ given that there are no current clinical guidelines for transitional care for TBI.

While the standards for rehabilitation facilities set by the Commission on Accreditation of Rehabilitation Facilities (CARF) helps to ensure that uniform guidelines are followed for discharge planning, people with TBI and their families identify the post-hospital transition as being a uniquely challenging time characterized by stress and uncertainty.^36^ For example, despite CARF guidelines, TBI Model Systems (TBIMS) data show that 28% of those with msTBI are rehospitalized within the first year of injury, and rehospitalization rates remain high up to 10 years post-injury.^37–39^ Rehospitalization, which is associated with reduced community participation after TBI,^40^ may be partly attributable to lack of transitional support and services that are appropriate for those with TBI.^38^ As the duration of acute and IRF hospitalization decreases steadily in the United States,^41,42^ modifiable contributors to suboptimal outcomes require careful consideration. For example, the recommendation to educate patients about home care almost immediately after hospital admission^43^ is problematic for individuals who are in post-traumatic amnesia (PTA) for all or most of their hospital stay and are thus unable to retain information. Important information provided to patients and their families at hospital discharge may also be poorly recalled due to its perceived lack of personal relevance, the timing of information provision, manner of teaching, patient health literacy and the emotional state of the patient/caregiver.^29^ Together, these findings suggest that enhanced discharge planning and extended transitional care services beyond the time of hospital discharge and available via telephone or other methods to provide information when it is needed may promote improved patient health and community participation during this critical transition period.

The Veterans Health Administration (VHA), workers’ compensation, other specialized insurance programs, and some state brain injury programs employ an intensive form of case management, a component of transitional care services, for complex populations, such as msTBI.^44–47^ Research in other clinical populations^48,49^ suggests that transitional care management can result in reduction of 30 days hospital readmission, as well as improved function and decreased health care utilization.^50,51^ Additionally, case management can support service linkage for populations’ most disadvantaged by structural barriers to accessing care. For TBI, systematic reviews of case management have provided mixed results, due in large part to inconsistent definitions of case management, inadequate comparators, low statistical power, selection bias, and varied follow-up time periods.^52,53^ A report to Congress from the Centers for Disease Control and Prevention called on the field to “develop and evaluate service models that will assist patients to better navigate the post-acute rehabilitation setting.”^2^

In view of these needs, the goal of this study was to compare the effectiveness of the CARF model, focused on transition planning prior to discharge (hereafter denominated Rehabilitation Discharge Plan [RDP]), with a more intensive approach, extended beyond discharge, to enhance discharge services and transitional care such as the one utilized in the VHA (denominated Rehabilitation Transition Plan [RTP]) as both are currently in practice but have not been previously studied.

The primary aim of this study was to compare the effectiveness of RDP versus RTP on improving patient-reported outcomes of 1. participation, as measured by the Participation Assessment with Recombined Tools-Objective 17 (PART-O-17)^54^ and 2. health-related quality of life (HRQoL), as measured by the Quality of Life after Brain Injury (QOLIBRI) scale,^55^ for individuals with msTBI who are discharged from inpatient rehabilitation.

The secondary aim was to compare the effect of the interventions on the trajectory of outcomes across the first-year post-discharge (with measurement at 3-, 6-, 9-, and 12-months post-discharge) on patient-reported outcomes of participation and HRQoL, while adjusting for other factors. Additionally, we explored several factors as possible moderators of the outcomes and explored the effect of the COVID-19 pandemic that occurred during the time of the study.

## Materials and Methods

The Brain Injury Rehabilitation: Improving the Transition Experience (BRITE) study was a six-center, 1:1 randomized pragmatic clinical trial with masked outcome assessment that compared the effectiveness of two established approaches to managing transition from IRF discharge to the next phase of care for individuals with msTBI. Procedures are detailed elsewhere and were informed by significant input from our patient, family, and professional partners.^56^ Randomization was stratified by center and discharge location (skilled nursing facility vs. discharge to home/community). We stratified on discharge location due to the relatively lower frequency of discharge to facility (22% across all 6 study centers in 2015) and the expectation that individuals discharged to facilities may have more severe TBI, more significant other injuries, social situations, and fewer opportunities for participation which could influence the outcomes. Early on, two of our sites enrolled more participants than could be managed by the TBI care manager (TCM) delivering the intervention at those sites. In April 2018 a sampling enrollment protocol was developed, with review and adjustment on a quarterly basis available to all sites if needed. A further revision in August 2018 based the formula on cumulative data rather than previous quarter data for a more accurate reflection of overall numbers.

The six centers were all National Institutes of Disability Independent Living and Rehabilitation Research (NIDILRR)-funded TBIMS centers:^57^ University of Washington, Indiana University School of Medicine/Rehabilitation Hospital of Indiana, MossRehab Hospital/Moss Rehabilitation Research Institute, Baylor Scott & White Institute for Rehabilitation, The Ohio State University Wexner Medical Center, and Mount Sinai Health System. TBIMS centers are largely urban, academic medical centers offering specialized inpatient and outpatient rehabilitation for msTBI; many serve a large suburban/rural catchment. All centers adhered to CARF standards for discharge and followed TBIMS practices for enrollment, tracking, and follow-up.^58^ Briefly, these include identification of eligible participants via medical records, approach and enrollment during inpatient rehabilitation, and follow-up at 1st, 2nd, 5th year, and every 5th years after injury. For the current study, we followed participants through 1-year post-discharge.

### Participants

Patients aged 18 years and older with msTBI requiring inpatient rehabilitation were recruited from February 2018 to August 2022. Eligible patient participants were identified by research staff through medical record review, using the TBI case definition of the TBIMS^59^ which is damage to brain tissue caused by an external mechanical force as evidenced by medically documented loss of consciousness or PTA due to brain trauma or by objective neurological findings that can be reasonably attributed to TBI on physical examination or mental status examination and at least one of the following criteria: 1. PTA >24 h; 2. Trauma-related intracranial neuroimaging abnormalities; 3. loss of consciousness exceeding 30 min (unless due to sedation or intoxication); or 4. Glasgow Coma Scale score in the emergency department of less than 13 (unless due to intubation, sedation, or intoxication).

Inclusion criteria included: English-speaking, at least 18 years of age, discharged to the community (e.g., home, adult family home) or facility (e.g., skilled nursing facility), completing their first comprehensive IRF stay post-TBI, and able to provide informed consent independently or via a legally authorized representative (LAR). Exclusion criteria were minimal due to the pragmatic trial and included being in law enforcement custody and having no anticipated access to a phone post-discharge.

### Procedures

Eligible patients who were deemed oriented through prospective screening were verified with clinical staff to have capacity to consent. If they were not oriented or did not have capacity consent was sought from an LAR. To manage caseload burden at high-volume centers, a random sampling algorithm was instituted 14 months into enrollment and was used at any center if enrollment exceeded the planned target enrollment for that center. Prior to March 2020, all enrolled individuals were randomized within 1 week of IRF discharge. In March 2020, in response to COVID-19-related research restrictions that stopped in-person enrollment in the hospitals, enrollment by telephone was initiated and permitted up to 3 months post-discharge.

After consent, patients were randomized in equal proportions to one of the comparators via computer-generated randomization by the data manager and the assigned group was sent via email to the TCM. Research assistants who collected the outcome data by phone were masked to group assignment. To provide data on the success of masking, data collectors were asked to guess each participant’s group assignment after each outcome assessment and report whether they believed they were unmasked.

While the main goal of the study was to compare the effects of RDP versus RTP on patient outcomes, we also invited caregivers (when available) to enroll in the study to assess the intervention effects on their own outcomes. Results for caregivers will be published in a separate article.

### Interventions

All participants received the elements of RDP, which consisted of the standard CARF elements of discharge planning, including: 1. Patient and family education about TBI, both general and individualized to each person’s symptoms and level of function, along with hands-on training for caregivers as needed, and education on medications and symptoms to monitor after discharge. 2. Written discharge instructions, with a follow-up plan that included recommended appointments with primary care, physical medicine and rehabilitation and outpatient therapies, and a medication list that was reviewed with the patient and family prior to discharge; and 3. A brief phone call from an inpatient care provider within 3 days post-discharge to identify any immediate problems.

RTP was based on the model successfully employed by the VHA, and was chosen based on literature supporting a more intensive, temporally extended, and coordinated approach for patients likely to experience significant and prolonged problems after injury.^47,60,61^ In addition to the elements of RDP, participants randomized to the RTP comparator were to receive up to 12 scheduled contacts from a TCM within 6 months post-discharge with additional contacts as needed and determined by the care plan. RTP was provided over a shorter time period when participants were enrolled after they had been discharged, but participants could still receive up to 12 scheduled contacts. All TCMs had a Master of Social Work degree or comparable experience in health care and/or social services, but they were not required to have pre-existing TBI expertise as they received training on both TBI as well as the health care environment and community resources at each center. Additional details on training and supervision of TCMs has been previously published.^56^ A total of 13 TCMs delivered RTP during the study and were supervised both by local study investigators and by the co-principal investigators (J.M.H., J.R.F.) in a monthly group supervision meeting. Initial fidelity checks were performed during TCM training with a formal review of recorded intervention contacts. Most contacts between TCMs and participants happened by telephone but could also occur via Health Insurance Portability and Accountability Act (HIPAA)-compliant videoconferencing through Zoom^®^, depending on participants’ preference. The TCMs attempted to reach participants to complete the RTP-scheduled contacts until: (1) the planned number of contacts was completed, (2) the participant declined or was deemed unable to complete the contacts, or (3) the RTP window for intervention lapsed 6 months following discharge.

The content of these contacts has been described previously;^56^ briefly, they included review of and assistance with implementing discharge plans and needs assessment^27,62^ related to medical follow-up, daily living, managing TBI symptoms, and return to activity. TCMs provided practical assistance and resource facilitation (a central evidence-based component of RTP^63^); TBI education; care coordination; and culturally tailored support. With participant permission, TCMs could communicate information to participants’ care providers. As the end of RTP approached, TCMs helped establish continuity of care by facilitating a warm hand-off to a provider (e.g., their primary care provider) and/or to a local brain injury organization.

### Outcomes measurement

With input from our patient and family research partners, two primary outcomes that were the most important to them were identified: (1) participation in the home and community, measured by the PART-O-17 instrument and (2) HRQoL, measured by the QOLIBRI. Measures were taken at 3-, 6-, 9-, and 12-months after discharge from inpatient rehabilitation care. Table 1 provides an overview of outcomes and covariates.

### Power analysis

At the time of the study design, QOLIBRI was the only HRQoL specifically designed for TBI patients, although it had not been studied enough to provide information for sample size calculation. PART-O-17 is a more established instrument, and we used information from previous studies to conduct power analyses. We expected to enroll a minimum of 300 individuals per year for 3 years (total *N* = 900). Data from a sample discharged from inpatient TBI rehabilitation without any intervention showed a mean of 1.51 (SD = 0.65) for PART-O-17 total average score (which has a range of 0–5) at 3 months follow-up and of 1.66 (0.72) at 9 months follow-up.^64,65^ Minimal detectable differences were 0.57 and 0.63 at 3 and 9 months, respectively. Using these data as a guide, we performed power analysis for samples sizes varying from 500 to 900 individuals, with effects sizes varying from 0.50 to 0.80, significance level of 0.05, a common standard deviation of 0.7 (the highest of the values above), for a *t*-test for independent samples, and allocation ratio 1:1. The results showed that the power of finding a difference in PART-O-17 between the two interventions, if one existed, was close to 1 under these scenarios. Given the very large sample size, we expected a high power to detect a difference in QOLIBRI also. Further details regarding power analysis are available from the authors upon request.

### Statistical analysis

The analysis plan conformed to the intent-to-treat principle. At the end of the study, participants not assessed at the end of intervention were considered as having missing outcome values. Reasons for missingness^66^ are difficult to ascertain, but we assumed that the data were missing at random, that is, the probability that data were missing did not depend on unobserved data but might have depended on observed data (such as intervention type, and age). Therefore, we considered the complete data analysis as the primary analysis and used multiple imputation as a sensitivity analysis, comparing the results of both analyses for differences. The analyses for aims related to longitudinal trajectory relied on statistical methods (mixed generalized linear models and latent class growth analysis) that are robust to missing data at individual time points and did not use imputed data.

Significance level was set to 0.05 for both coprimary outcomes without correction, since the sample size and consequent statistical power were very high. For analyses of trajectory and heterogeneity of treatment effects, significance level was also set to 0.05 without correction, but these analyses are of exploratory nature and will be interpreted as preliminary. A stringent significance level might prematurely eliminate factors that should be studied further in future studies.

For the primary aim, both the PART-O-17 and the QOLIBRI are numeric with a fairly large range and were considered as continuous for data analysis. We tested the difference in means for PART-O-17 and QOLIBRI between the two groups at 6 months post-discharge using a two-sided *t*-test for independent samples, assuming unequal variance. We tested if the intervention had a long-term effect by comparing the two groups at 12 months post-discharge using the same type of *t*-test. These analyses were performed using SPSS and STATA with complete data and imputed data (50 imputed datasets).

To compare the trajectory of outcomes for 3-, 6-, 9-, and 12 months post-discharge, we plotted individual trajectories over time (outcome at each point in time for each participant) and used a linear mixed model, with the outcome (PART-O-17 or QOLIBRI) as the response variable; time and other factors (such as center, age, sex, race/ethnicity, education, and injury severity) as explanatory variables; and added intervention group and its interaction with time to test whether the interventions and time interacted to produce an effect on the trajectory of the outcome. To complement this approach, we also performed latent class growth analysis to classify participants into groups with similar trajectories for the outcome (PART-O-17 or QOLIBRI) and investigated whether treatment group membership (RTP vs. RDP) differed across trajectory class membership. The same procedure was used to examine differences in demographic and injury characteristics across trajectories.

The latent class growth models were performed in R using the *lcmm* package and included the follow-up time as a covariate and a random intercept for each participant and assumed the outcomes are a Gaussian mixture model.^67,68^ Bayesian information criterion was used to determine the model with the optimal number of classes. Fifty repetitions of each model were conducted using random starting values, to ensure the final model represented a global optimization.

#### Heterogeneity of treatment effects.

We explored the differential effects of the intervention on patient subgroups—or heterogeneity of treatment effects—defined by factors known or hypothesized to have associations with TBI outcome: facility versus community discharge, race/ethnicity, age, sex, severity of TBI (as measured by time in PTA), presence of preinjury psychosocial limitations (e.g., unemployment, substance abuse) and medical or psychiatric comorbidity, as well as study center and insurance type. One linear regression model was created for each factor, with the factor, the intervention group, and an interaction between the factor and the intervention group in the model, using PART-O-17 or QOLIBRI as the response variables. Based on previous research,^69–74^ we hypothesized that severity of TBI and presence of preinjury psychosocial limitations would interact with the intervention group.

#### COVID-19 impact.

Given the unexpected development of the COVID-19 pandemic during the current trial, we conducted a *post hoc* analyses to examine whether there were any differences related to the COVID-19 by analyzing: 1. the patterns and causes of refusal to participate in the study before and during the pandemic to determine if the populations in the two periods were coming from the same base population; 2. the patterns of missing data, comparing missing data before and during the pandemic to determine whether we needed to additionally add an indicator of the time of data collection (before or during pandemic) as another factor in the generation of the imputed outcome data; and 3. the primary outcomes in an intention-to-treat manner as proposed in the protocol. COVID-19-related time in the study was categorized as: those who enrolled and were 12 months post-discharge prior to the pandemic; those who enrolled before the pandemic and hit 12 months post-discharge after the pandemic commenced; and those who started and completed the study entirely during the pandemic. This categorization was included in a linear regression model as a factor, in addition to intervention type and the interaction between COVID-19-related time in the study and intervention, to explore the potential impact on the outcomes of the time period of participation in the study and the intervention delivered during the pandemic.

## Results

**FIG. 1.**
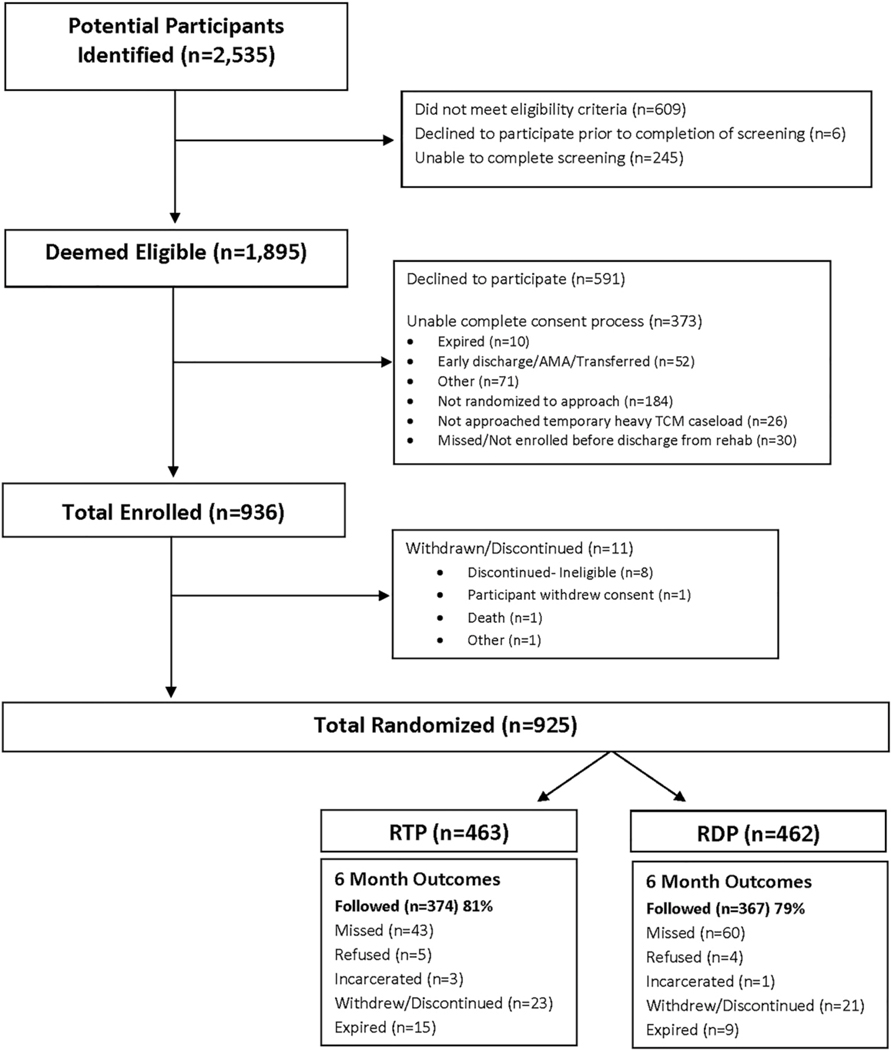
Final consort diagram, Brain Injury Rehabilitation: Improving the Transition Experience (BRITE) study: patient participants.

Between February 2018 and August 2021, a total of 1926 patients met inclusion criteria, and a total of 925 patients were randomized. Figure 1 shows the flow of participants through the study from screening through all outcome assessments.

Participants had a mean age of 47 years, about 73% were males, and just under 67% were Whites. Refer to Table 2 for a description of the sample.

A total of 741 participants completed an outcome assessment at the 6 months primary outcome time point (374 [81%] in the RTP group and 367 [79%] in the RDP group). See Figure 1 for details on attrition and Supplementary Table S1 for baseline differences between those who completed and those who were missing due to death or other reasons. Results suggest that those with missing data were older, less likely to be married, more likely to be injured by a fall or violence, be discharged to a facility, have more preinjury limitations, and lower motor and cognitive function at discharge from inpatient rehabilitation. They were also less likely to have a caregiver enrolled in the study and more likely to have started in the study prior to the pandemic and finish during the pandemic.

Data collectors accurately guessed the participants’ assigned treatment group nearly twice as frequently for the RDP than for the RTP group (75% vs. 39%, with 50% expected by chance alone). In contrast, masked staff reported being unmasked only 7% of the time, with the majority of reported unmasking occurring in the RTP arm (mostly when a participant made a reference about the TCM during the outcome assessment interview, despite being reminded not to discuss any treatment contacts).

Of 463 participants randomized to RTP, 74 (16%) did not complete the intervention, with 28 not having any contact with a TCM (<1 call; at least 9 of whom where no contact was initiated due to TCM staffing issues), 24 withdrew or discontinued, 13 died prior to the completion of the RTP intervention (6 months post-discharge) and an additional 8 were lost for other reasons, and 1 who had an adverse event unrelated to the study.

### Primary aim

To compare the effectiveness of RDP versus RTP on improving patient-reported outcomes of participation and HRQoL. Table 3 shows the results for both outcomes at 6- and 12-month post-discharge follow-ups. We found no differences between RTP and RDP for measures of participation (PART-O-17) and quality of life (QOLIBRI) at 6- and 12-months’ follow-ups, either with complete or with imputed data (not shown here as results did not differ significantly).

### Secondary aim

To compare the trajectory of improvement across the first-year post-discharge on patient-reported outcomes of participation and quality of life. The final *linear mixed model* for PART-O-17 showed no statistical significance for the intervention or for the interaction between intervention and time. The resultant model showed that PART-O-17 scores increased with increasing follow-up time, discharge Functional Independence Measure (FIM) motor and FIM cognitive scores, and education level (compared to less than high school), with decreasing age, and for individuals discharged to the community (compared to facility), with private or other insurance (compared to Medicare), with no prior limitations (compared to at least one prior limitation), and having started and finished the study prior to the pandemic. Since center was statistically significant, we calculated the expected change in PART-O-17 relative to group RTP in site C, when all the other variables are the same, which are shown in the last column of Table 4. Two centers (E and F) had consistently lower participation scores, while two others (A and B) had consistently higher scores for both interventions when compared to Center C.

**FIG. 2.**
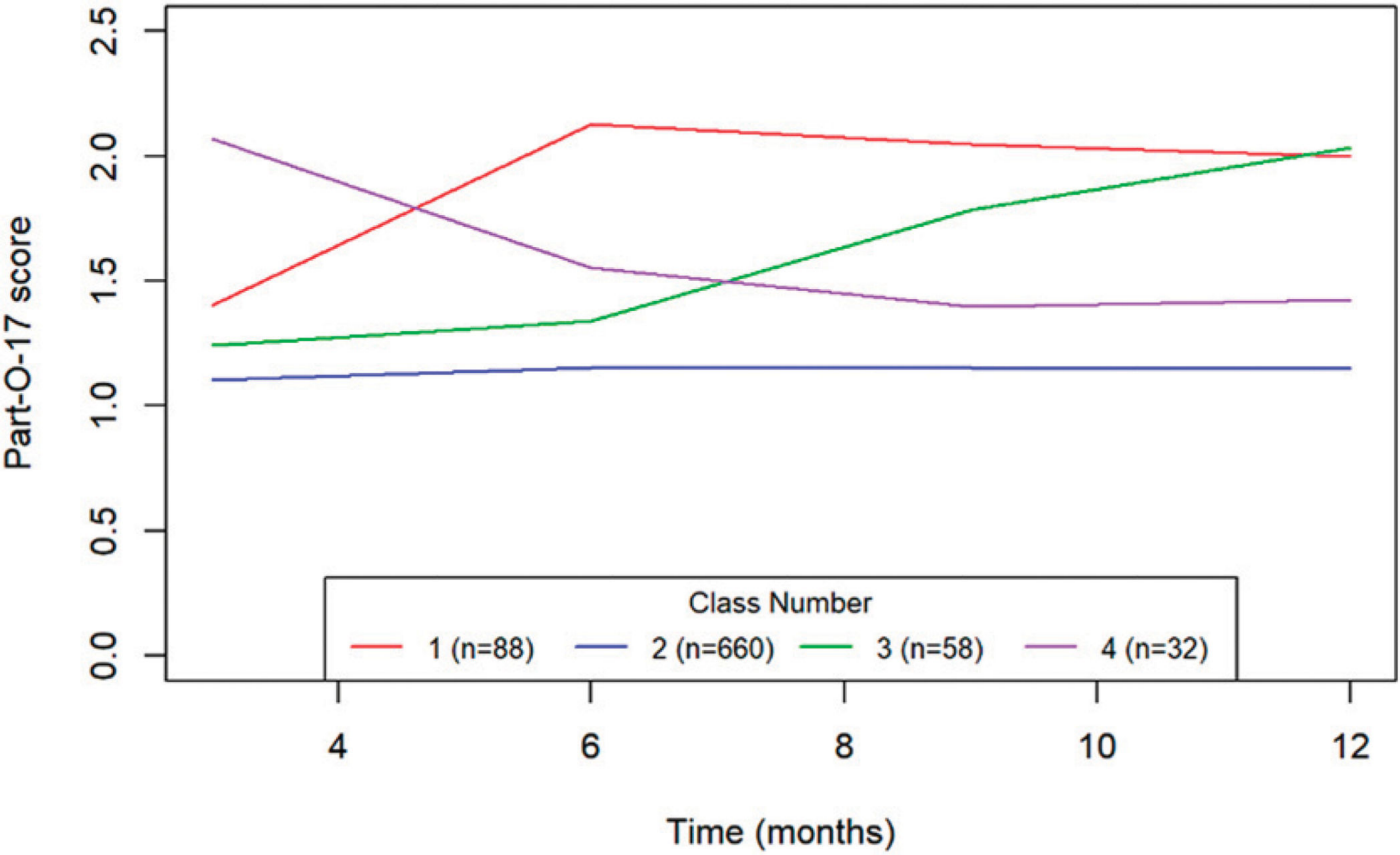
Latent growth curve model for Participation Assessment with Recombined Tools-Objective (PART-O).

Using the *latent growth model*, the model with four different trajectories of participation (PART-O-17) best fit the data (see BIC from each model in Fig. 2). The four distinct trajectory classes (illustrated in Fig. 2) are: 1. those who had an immediate increase in PART-O-17 and then plateaued; 2. those with low PART-O-17 initially and little change; 3. those who had a delayed increase in PART-O-17; and 4. those who had a decrease in PART-O-17. The majority of participants, regardless of treatment assignment, were in class 2. No clear pattern between trajectory group and treatment group was found.

Smaller sample sizes were available for the QOLIBRI, since the instrument cannot be completed by a proxy, thus excluding participants that were not able to answer the instrument by themselves. Models for quality of life as measured by QOLIBRI were constructed as described above. Time, intervention, center, and the interaction between center and intervention were not statistically significant in any of the models, including the final one. Quality of life as measured by QOLIBRI, on average, increased with increasing follow-up time (but not significantly), FIM motor, FIM cognitive, and education level (compared to less than high school), with decreasing age, and for males (compared to females), individuals living alone, with no prior limitations (compared to at least one prior limitation), and who never received mental health care. Also, Medicare-insured individuals had higher estimated QOLIBRI, followed by other, private/self, and Medicaid (all other variables being the same).

**FIG. 3.**
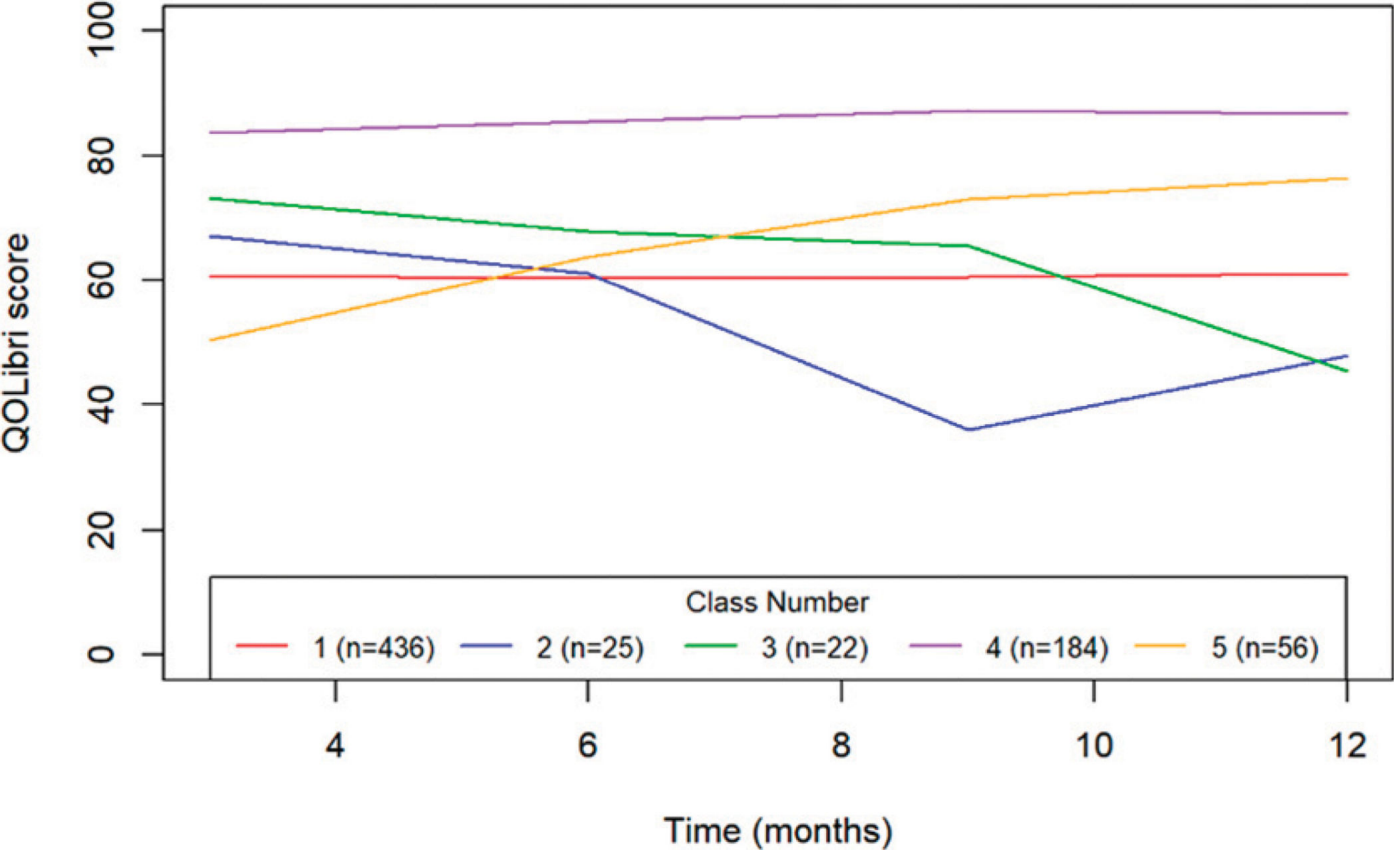
Latent growth curve model for Quality of Life after Brain Injury (QOLIBRI) scale.

The latent growth model analysis found five trajectories best fit the QOLIBRI data (see Fig. 3): (1) low scores with little change; (2) a large decrease in scores from 6 to 9 months; (3) a large decrease in scores from 9 to 12 months, (4) high scores with little change; and (5) steady increase in scores. The majority of participants fell into groups 1 or 4, and experienced little change throughout the study period, regardless of treatment assignment, although those in group 4 had consistently higher scores than the other groups. No clear pattern between trajectory group and treatment group was found.

### Heterogeneity of treatment effects

We examined differential effects of interventions across factors known to be associated with TBI outcome (see Table 5 for a list of variables and summary of findings) at 6- and 12-months for both participation and quality of life outcomes. All results should be interpreted with caution and considered preliminary, given the multiple comparisons conducted. Overall, there were only three interaction effects of interventions with other variables. At 6 months, individuals with no caregiver in the RTP group had much higher participation than those with caregivers, while the difference was very small in the RDP group. At 6 months, having a caregiver was associated with higher quality of life if a participant was in the RDP group, but not having a caregiver was associated with higher quality of life for those in the RTP group. In addition, at 12 months, there was a significant group by center interaction, where two centers had greater participation in the RDP group and four centers had greater participation in the RTP group. Complete results can be seen in Supplementary Tables S2, S3, S4, and S5.

Beyond the three interactions, results suggest that there was a significant difference by center for both outcomes (participation and quality of life at 6- and 12-months; see Table 5). In addition, presence of preinjury limitations, having Medicaid insurance (instead of Medicare or Private/other), and lower cognitive and motor function were associated with worse participation and quality of life at both time points. Increasing age was associated with worse outcomes at 6 months and lower participation at 12 months as well, but not quality of life at 12 months. Discharge to a facility was associated with worse participation at 6- and 12-months post-discharge but not associated with quality of life. Males reported better quality of life at 6- and 12-months than females, but sex was not associated with participation. No other consistent differences were seen.

### COVID-19 impact

Individuals randomized into the study were classified according to the time in the study relative to the COVID-19 pandemic. Full analyses can be found in Supplementary Appendix. Overall, there appeared to be limited impact of COVID-19 on participation and quality of life (see Table 5), and there were no differences related to the impact of the intervention.

## Discussion

In this pragmatic trial, we compared two approaches for the transition from IRF to home/community or facility following msTBI, one consisting of transition planning prior to rehabilitation hospital discharge, and the other providing transition planning plus scheduled ongoing contacts for needs assessment and case management over the 6 months following discharge. We found no evidence of a difference between the two interventions on the primary outcomes, societal participation, and HRQoL, outcomes that had been selected with input from patient and family partners involved in the research. Likewise, analysis of trajectory of patient reported outcomes utilizing latent growth models did not show treatment group differences, and most patient participants were found in groups that had minimal change across all time points. There was also no evidence of any consistent impact of the COVID-19 pandemic on outcomes.

Results of analyses examining heterogeneity of treatment effects suggest that at 12 months post-discharge, four centers had significantly higher PART-O-17 scores in the RTP than RDP while the other two centers showed opposite results. At 12 months post-discharge, those living in rural areas had higher participation than those in urban/suburban areas, but without differential treatment effects. Fewer heterogeneity factors were associated with quality of life as measured by the QOLIBRI, but there were differences observed across centers. Those with a history of preinjury limitations and older participants also had lower quality of life. At 12 months, those with lower cognitive and motor function at discharge endorsed lower quality of life.

Overall, our hypotheses regarding the superiority of the RTP model compared to the CARF-compliant RDP approach were not supported. There are several possible reasons for these findings. First, the primary outcome measures may not have been sufficiently sensitive to the effects of the RTP intervention. The patient-reported outcomes of participation and quality of life were selected for their importance to our patient and family partners, but the intervention had not been specifically targeted to enhance these outcomes. Although scores on these measures varied across participants as demonstrated in latent class growth trajectories, these outcomes did not change across the first-year post-injury for most participants in the current sample. TCM support may have yielded more specific health-related benefits such as greater access to care or connection with needed resources, which will be explored in future analyses.

Related to this point, participants may have benefited from the RTP intervention in ways not measured in this study. For example, participants may have felt less anxious, more supported, or more confident in their recovery when receiving regular contacts after leaving the hospital. While we did not specifically measure these or other mental health-related effects, a survey conducted with RTP participants provided indirect support for these alternative outcomes. Results suggested the intervention was well-received and would be highly recommended to others, which suggests that it may act on unmeasured outcomes that may be mechanistically related to longer-term outcomes.^76^ It is therefore possible that community participation and HRQoL should be considered “distal” outcomes of the treatment offered in this investigation, as change may be observed only after most urgent needs are met. Participation and life quality are broad outcomes likely to be affected by many external factors beyond the intervention, and changes in such outcomes may not flow directly or solely from the tested transitional care approaches.

Second, the modifications to the VHA model that we made to optimize feasibility and scalability (e.g., contacts limited to 6 months) may have diluted its effectiveness, especially given relatively short lengths of stay during inpatient rehabilitation where more acute and complex issues may need to be addressed post-discharge. Moreover, effective resource facilitation requires the availability of resources. In contrast to the VHA, there is often a paucity of resources available for civilians with msTBI upon discharge to the community. While outpatient rehabilitation therapies are commonly available, services and supports to assist with behavioral health problems, financial difficulties, housing insecurity, or other psychosocial issues are sorely lacking in the US communities.^26^ Transitional case management is intended to guide persons to available resources but cannot compensate for the absence of supports and services that could be capable of changing participation and quality of life. In addition, while we attempted to identify community resources across all categories of need identified by participants, these resources changed throughout the course of the study. This was especially complex during the COVID-19 pandemic when resources such as adult day programs, for example, were closed and some did not reopen again.

Finally, an observation by TCMs that was repeatedly discussed during group and site-level supervision meetings was that some individuals randomized to the RTP group did not seem to want or view themselves as needing post-discharge support. In some cases, this included individuals deemed to have high needs per needs assessments, input from caregivers, or TCM observation. The discrepancy between clinician and participant perceptions of need may reflect participants’ personal preferences, existence of extensive resources and supports that obviated the need for RTP, or lack of insight into injury-related deficits and how they may impact functional independence, self-care, and ability to access and utilize available resources. Repeated TCM contacts may have been perceived as intrusive by those who do not need, or who do not believe themselves to need, this support. Given the study randomization scheme, there is no reason to believe that any of these factors differed across treatment groups. The possibility that an intervention such as RTP may be most appropriately deployed to those deemed to need and/or want it, as is suggested by recommendations to align post-TBI interventions with individual needs,^77^ warrants further investigation.

Regarding the broader implications for telerehabilitation in msTBI, the lack of impact of a global pandemic on current findings, together with the ability of the study team to continue the RTP intervention during the pandemic, speaks to the feasibility and scalability of remote TCM interventions. The ability to reach individuals and provide care via telemedicine that was quickly put into place during the pandemic may have reduced negative impacts for those who traditionally have difficulty traveling to hospitals or free-standing clinics for care. Future research should consider monitoring the availability of telemedicine which, as of this writing, is being reduced given lack of payment (facility-fee) for hospital-based clinics which are shifting back to in-person visits to meet budget.

Our results are likely generalizable to patients who received care from CARF-accredited IRFs, but perhaps not to patients who do not receive care from a CARF-accredited and/or TBIMS facility. Current standards for admission to IRF can be limited by insurance coverage, perceived tolerance of intensive rehabilitation therapies, and having a stable discharge location identified prior to admission. Given these restrictions, our participants may have had natural supports which we did not fully appreciate or assess. In addition, it is possible that the TBIMS status of all participating centers could affect generalizability (e.g., floor effect’); there could be unmeasured differences between the follow-up care offered at the specialized TBIMS centers versus other CARF-accredited facilities that treat similar patients. Arguing against this is the fact that stakeholders from our clinical programs clearly endorsed a need for greater care coordination and support post-discharge. Nonetheless, it might be instructive to examine the RTP model in clinical settings that offer shorter or less intensive in-person support to people with msTBI or in those deemed ineligible for inpatient rehabilitation admission.

As we had anticipated, there were several factors that contributed to heterogeneity of outcomes, including differences across clinical sites. Further investigation is needed to better understand the sources of site differences: demographic and injury characteristics among treated patients, or the type and number of resources available in the community. There may have been unmeasured differences in the delivery of RTP, despite our efforts to standardize treatment. Even in a pragmatic trial, which is designed to accommodate real-world variation, it would have been instructive to conduct more detailed measurement of the treatment so as to analyze its impact on outcomes.

### Limitations

The current study design precluded identification of a “dose” of the intervention given that the RTP was highly person-centered. For example, one participant may have received the full 12 calls as they had many needs to be addressed and another may have also received the full 12 calls even after returning to work with fewer needs. In addition, we did not measure actual uptake or engagement with resources. Future research should consider methods to capture these differences in experience. Also, we limited examination of heterogeneity of treatment effects to single factors rather than examination of potential intersectional groups due to sample size where much larger data sets would be needed in order to help identify those most in need of an enhanced transition process or who might be more responsive to intervention. Future work should consider including large numbers of historically underrepresented groups to determine whether a different version of RTP might be beneficial to them.

Our intervention was limited to the first 6 months post-discharge from IRF, which our patient, family, and professional partners reported might be too short for the many needs that evolve after msTBI. The heterogeneity of outcomes and their time frame, the fact that insight into problems may only develop later in the recovery process, and the gradual exhaustion of family and clinical support are some of the reasons that a longer period of support might be beneficial. Unfortunately, milestone-driven timelines of studies with time-limited funding make it difficult to study longer term interventions.

We are unable to determine whether the two approaches to transition from IRF to the community lacked sufficient intensity to lead to a change in community participation and/or HRQoL or whether the measures that we used were unable to detect an actual change in these outcomes. Given that there was a lack of change in both outcomes over time, further research is needed to address this limitation. In addition, we did not collect detailed information on site differences or the range of co-occurring conditions that could add complexity and that may have masked potential effectiveness of the intervention.

## Conclusions

The principal aim of the BRITE study was to compare the effectiveness of two existing approaches to the transition of care between inpatient rehabilitation and the community for those with msTBI. Transition from inpatient rehabilitation is a potential important point of intervention for those with msTBI and their families to learn about the chronic nature of msTBI and how to connect with resources in the community. Intention-to-treat analysis did not show significant differences in primary outcomes of participation and HRQoL for patients across the first year after hospitalization. Further exploration of the data may provide insight into whether there are intersectional groups who may have benefited from each approach and whether there are other measurable outcomes better suited to testing the intervention.

Although we did not demonstrate superiority of a 6-month-long transitional support program (RTP), results from this study generated many more questions that should be addressed in future research. These include determining RTP treatment-responsive outcomes, as well as investigating how those outcomes may influence subsequent participation and quality of life; when and for how long to administer such interventions; identifying who most benefits from RTP or other interventions; and how a follow-up transitional care program should be modified to best suit the needs of individuals impacted by msTBI.

### Transparency, Rigor, and Reproducibility Statement

The study design and analysis plan were preregistered on February 5, 2018 at Clinicaltrials.gov (NCT03422276). Prespecified sample size was 450 per group, yielding statistical power across all outcomes of 80% for detection of an effect size of at least 0.3. All subjects were assigned to their intervention arm (RDP vs. RTP) using a random number generator stratified by discharge location (community vs. facility), yielding groups that did not differ in baseline characteristics. A total of 936 subjects were enrolled and 925 were randomized and primary outcomes were assessed in 741 subjects after 24 deaths, 103 missed assessments, 9 refused, 4 incarcerated, and 44 withdrew. All primary outcomes were assessed by research staff blinded to group assignment and guessed the group assignment of RDP at a higher rate than RTP. The primary outcome measures are validated and used in TBI. Sensitivity analyses were conducted using imputation. The findings have not yet been replicated or externally validated. Data and analytic code have been deposited at ICPSR (Inter-university Consortium for Political and Social Research) and will become available upon request.

## Supplementary Material

Supplemental Table 4

Supplemental Table 5

Supplemental Appendix

Supplemental Table 3

Supplemental Table 2

Supplemental Table 1

Supplementary Appendix

Supplementary Table S1

Supplementary Table S2

Supplementary Table S3

Supplementary Table S4

Supplementary Table S5

## Figures and Tables

**Table 1.** Study Outcomes, Covariates, and Data Sources

Outcomes/covariates	Data source(s)	When	How assessed

Primary outcomes			
Participation in usual roles and activities	Patient participant or appropriate proxy	3-, 6-, 9-, and 12-months post-discharge	Participation Assessment with Recombined Tools-Objective 17^63^
Health-related quality of life	Patient participant	3-, 6-, 9-, and 12-months post-discharge	Quality of Life after Brain Injury Scale^64^
Covariates			
Discharge location	Medical record	Time of discharge	Abstracted and coded as community or facility
Patient demographic characteristics	Self-report, proxy, medical record	Baseline	Age, sex, race, education, marital status, employment, earnings, language, residence
Substance use	Self-report	Baseline; 1-year post-injury	TBIMS preinjury and Form II tobacco and alcohol use questions
Prior limitations	Self-report or proxy	Baseline	TBIMS preinjury prior limitations questions
Medical or psychiatric comorbidity	Self-report, proxy, or medical record	Baseline; 1-year post-injury	TBIMS preinjury medical and psychiatric questions; TBIMS Form II NHANES health conditions
Medical insurance	Self-report; medical record	Baseline; 6- and 12-months post-discharge	Primary rehab payor; current medical insurance at follow-up

TBIMS, Traumatic Brain Injury Model System; NHANES, National Health and Nutrition Examination Survey.

**Table 2.** Description of the Sample as Randomized

Characteristics	Rehab transition plan	Rehab discharge plan	Total sample

Sample Size	463	462	925
Study Center, *n* (%)			
Site A	83 (17.9)	82 (17.7)	165 (17.8)
Site B	75 (16.2)	76 (16.5)	151 (16.3)
Site C	70 (15.1)	72 (15.6)	142 (15.4)
Site D	86 (18.6)	86 (18.6)	172 (18.6)
Site E	51 (11.0)	49 (10.6)	100 (10.8)
Site F	98 (21.2)	97 (21.0)	195 (21.0)
Sociodemographic characteristics			
Age in years, mean (SD)	47 (20)	48 (20)	47 (20)
Median (min., max.)	46 (18, 90)	47 (18, 90)	47 (18, 90)
Sex, *n* (%) of males	349 (75.4)	328 (71.0)	677 (73.2)
Education level, *n* (%)			
Less than high school	59 (12.9)	66 (14.5)	125 (13.7)
High school diploma or GED	152 (33.3)	161 (35.5)	313 (34.4)
Some college	118 (25.8)	126 (27.8)	244 (26.8)
College or more	128 (28.0)	101 (22.2)	229 (25.1)
Missing values, *n*	6	8	14
Married/with partner, *n* (%) yes	189 (41.3)	159 (34.7)	348 (38.0)
Missing values, *n*	5	4	9
Smoker^a^, *n* (%) yes	163 (35.7)	134 (29.3)	297 (32.5)
Missing values, *n*	6	5	11
Race, *n* (%)			
White	314 (68.7)	296 (64.6)	610 (66.7)
Black	79 (17.3)	81 (17.7)	160 (17.5)
Hispanic	33 (7.2)	53 (11.6)	86 (9.4)
Other	31 (6.8)	28 (6.1)	59 (6.4)
Missing values, *n*	6	4	10
Hispanic ethnicity, *n* (%) yes	29 (6.4)	47 (10.5)	76 (8.4)
Missing values, *n*	11	13	24
Competitively employed^a^, *n* (%) yes	284 (61.9)	268 (58.3)	552 (60.1)
Missing values, *n*	4	2	6
Annual earnings^a^, *n* (%)			
None	155 (37.4)	167 (40.0)	322 (38.7)
Less than 30K	84 (20.3)	84 (20.1)	168 (20.2)
30K to less than 60K	89 (21.5)	87 (20.8)	176 (21.2)
60 K or more	86 (20.8)	80 (19.1)	166 (20.0)
Missing values, *n*	49	44	93
English spoken at home, *n* (%) yes	428 (93.4)	433 (94.3)	861 (93.9)
Missing values, *n*	5	3	8
Residence zone of discharge			
Urban/suburban, (*n* (%)	301 (66.4)	314 (69.3)	615 (67.9)
Rural, *n* (%)	152 (33.6)	139 (30.7)	291 (32.1)
Missing values, *n*	10	9	19
Clinical characteristics			
Ever used illicit drugs^a^, *n* (%) yes	120 (26.4)	102 (22.6)	222 (24.5)
Missing values, *n*	9	10	19
Ever had psychiatric hospitalization^a^, *n* (%) yes	40 (8.8)	39 (8.6)	79 (8.7)
Missing values, *n*	6	6	12
Ever had mental health treatment^a^, *n* (%) yes	136 (29.8)	112 (24.6)	248 (27.2)
Missing values, *n*	6	6	12
Ever attempted suicide^a^, *n* (%) yes	35 (7.7)	25 (5.5)	60 (6.6)
Missing values, *n*	7	10	17
Ever served in military^a^, *n* (%) yes	60 (13.1)	55 (12.0)	115 (12.6)
Missing values, *n*	5	5	10
Living alone at discharge, *n* (%) yes	15 (3.3)	23 (5.0)	38 (4.1)
Missing values, *n*	4	3	7
Discharge to facility, *n* (%) yes	89 (19.2)	89 (19.3)	178 (19.2)
SCI, *n* (%) yes	58 (12.7)	58 (12.6)	116 (12.7)
Missing values, *n*	6	3	9
Limitations prior injury^a^, *n* (%) yes	209 (45.6)	222 (48.6)	431 (47.1)
Missing values, *n*	5	5	10
Alcohol use problem^a^, *n* (%) yes	97 (22.2)	111 (25.9)	208 (24.0)
Missing values, *n*	26	34	60
Injury-related characteristics			
Cause of injury, *n* (%)			
Vehicle (includes pedestrian/bike)	248 (54.3)	232 (50.9)	480 (52.6)
Fall	146 (31.9)	168 (36.8)	314 (34.4)
Violence	41 (9.0)	40 (8.8)	81 (8.9)
Other	22 (4.8)	16 (3.5)	38 (4.2)
Missing values, *n*	6	6	12
Rehab length of stay, days, mean (SD)	26 (26)	26 (23)	26 (24)
Median (min., max.)	18 (3,264)	19 (2, 172)	18 (2, 264)
Missing values, *n*	4	3	8
FIM cognitive at discharge, mean (SD)	22.9 (6.7)	23.0 (6.7)	23.0 (6.7)
Median (min., max.)	24.0 (5.0, 35.0)	24.0 (5.0, 35.0)	24.0 (5.0, 35.0)
Missing values, *n*	57	43	100
FIM motor at discharge^b^, mean (SD)	64.1 (19.7)	63.3 (20.0)	63.7 (19.8)
Median (min., max.)	68.0 (12.8,91.0)	66.0 (12.8,91.0)	67.0 (12.8, 91.0)
Missing values, *n*	8	7	15
Insurance type, *n* (%)
Medicare	98 (21.4)	118 (25.7)	216 (23.5)
Medicaid	118 (25.7)	108 (23.5)	226 (24.6)
Private/self	199 (43.4)	180 (39.2)	379 (41.3)
Other, workers comp, charity	44 (9.6)	53 (11.5)	97 (10.6)
Missing values, *n*	4	3	7
GCS severity score, *n* (%)			
Severe (3–8)	65 (15.6)	55 (12.9)	120 (14.2)
Moderate (9–12)	42 (10.1)	41 (9.6)	83 (9.8)
Mild (13–15)	127 (30.5)	159 (37.2)	286 (33.9)
Sedated/intubated	182 (43.8)	172 (40.3)	354 (42.0)
Missing values, *n*	47	35	82
Severity according to time in PTA			
0–24 h (mild)	70 (16.8)	73 (17.4)	143 (17.1)
25 h to 7 days (moderate)	47 (11.3)	43 (10.2)	90 (10.8)
More than 7 days (severe)^c^	299 (71.9)	304 (72.4)	603 (72.1)
Missing values, *n*	47	42	89
Other characteristics			
Caregiver enrolled in the study, *n* (%) yes	293 (63.3)	292 (63.2)	585 (63.2)
Time in the study relative to COVID-19, *n* (%)			
Completed study prior to pandemic	136 (29.4)	134 (29.0)	270 (29.2)
Started pre-COVID, ended during pandemic	129 (27.9)	136 (29.4)	265 (28.6)
Started and ended during pandemic	198 (42.8)	192 (41.6)	390 (42.2)

Missing values were removed before calculating percentages in each group.

a These characteristics refer to time prior to injury.

b FIM Motor was either collected with FIM instrument or was transformed from the CARE tool to FIM Motor, according to the crosswalk developed by Dave Mellick.^75^

c Includes individuals who were discharge still in PTA.

GED, General Educational Development; SCI, spinal cord injury; FIM, Functional Independence Measure; GCS, Glasgow Coma Scale; PTA, post-traumatic amnesia; SD, standard deviation.

**Table 3.** Results from Data Analysis of BRITE Outcomes at 6- and 12-Months Follow-Ups with Complete Data

Outcome	*Data used*	*Sample size*	*Observed means (SD)*	*t*	p *value*

PART-O-17 at 6M					
	Complete (n = 735)	RTP = 372 RDP = 363	RTP = 1.33 (0.57) RDP = 1.30 (0.61)	0.78	0.44
PART-O-17 at 12M					
	Complete (n = 713)	RTP = 354 RDP = 359	RTP = 1.35 (0.58) RDP = 1.36 (0.61)	0.37	0.71
QOLIBRI at 6M					
	Complete (n = 606)	RTP = 315 RDP = 291	RTP = 65.9 (17.8) RDP = 68.3 (19.3)	1.63	0.10
QOLIBRI at 12M					
	Complete (n = 595)	RTP = 292 RDP = 303	RTP = 65.8 (19.3) RDP = 68.3 (20.2)	1.59	0.11

PART-O-17, Participation Assessment with Recombined Tools-Objective 17; RTP, Rehabilitation Transition Plan; RDP, Rehabilitation Discharge Plan; QOLIBRI, Quality of Life after Brain Injury Scale.

**Table 4.** Expected Change in PART-O-17 Outcome by Interaction of Intervention and Center (All Other Variables Being the Same)

	Intervention	Center	Center × intervention	Expected change in Part-O-17 compared to individuals in C and RTP

RTP				
A	0	0.0454666	0	0.045
B	0	0.1858463	0	0.186
C	0	0	0	0
D	0	−0.0400485	0	−0.040
E	0	−0.1713988	0	−0.171
F	0	−0.0430345	0	−0.044
RDP				
A	0.0259196	0.0454666	0.0959675	0.167
B	0.0259196	0.1858463	−0.113965	0.098
C	0.0259196	0	0	0.026
D	0.0259196	−0.0400485	0.0232278	0.009
E	0.0259196	−0.1713988	−0.0617332	−0.207
F	0.0259196	−0.0430345	−0.0419075	−0.059

PART-O-17, Participation Assessment with Recombined Tools-Objective-17; RDP, Rehabilitation Discharge Plan; RTP, Rehabilitation Transition Plan.

**Table 5.** Summary of Heterogeneity Analyses by Outcome and Time

	Outcome and time period			
Explanatory variable	PART-O-17 at 6 months	PART-O-17 at 12 months	QOLIBRI at 6 months	QOLIBRI at 12 months

Center	a	b a	a c	a
Presence of prior limitations	a	a c	a	a
Type of insurance (Medicare; Medicaid; Private, other)	a	a	a c	a
FIM cognitive at discharge	a	a	a	a c
FIM motor at discharge	a	a	a	a
Age	a	a	a	d
Discharge to facility or community	a	a	a	d
Sex	d	d	a	a c
Having an enrolled caregiver	b a	d	b	d
Race (4 categories)	a c	d	d	d
Rural versus urban/suburban	d c	a	d	d
PTA severity	d	d	d	d
COVID-19 period	a	d	d	d

a Variable statistically significant at 0.05 level.

b Interaction between intervention group and variable present in the complete data model.

c Result for imputed data analysis differs from results for complete data analysis shown here.

d Variable not statistically significant.

PART-O-17, Participation Assessment with Recombined Tools-Objective-17; RDP, Rehabilitation Discharge Plan; RTP, Rehabilitation Transition Plan; QOLIBRI, Quality of Life after Brain Injury (QOLIBRI) scale; FIM; Functional Independence Measure; PTA, post-traumatic amnesia.
