# Supplementary material for: Comparing Two Models of Transition from Inpatient Rehabilitation Following Traumatic Brain Injury: A Pragmatic Comparative Effectiveness Trial
Source: J Neurotrauma. Author manuscript; Available in PMC 2026 Jun 25. (PMC13296878; doi:10.1177/08977151251374298)
Supplement: Supplemental Table 4 [file NIHMS2162225-supplement-Supplemental_Table_4.docx]

**Supplemental Table 4. Results of heterogeneity analysis for PART-O-17 at 12-month follow-up**

| **Explanatory Variable** | **Data used** | **Sample Size** | **P-value for** | **Intervention Group Estimated Means (SE)** | |
| --- | --- | --- | --- | --- | --- |
|  |  |  |  | **RTP** | **RDP** |
| Center | Complete  (n=713) | RTP=354  RDP=359 | Group: 0.94  Center: <0.001  Interaction: 0.04 | Site A: 1.37 (0.07)  Site B: 1.54 (0.08)  Site C: 1.38 (0.08)  Site D: 1.34 (0.07)  Site E: 1.20 (0.10)  Site F: 1.20 (0.06) | Site A: 1.60 (0.07)  Site B: 1.43 (0.08)  Site C: 1.35 (0.09)  Site D: 1.50 (0.06)  Site E: 0.97 (0.08)  Site F: 1.16 (0.07) |
| PTA severity | Complete  (n=649) | RTP=321  RDP=328 | Group: 0.38  Severity: 0.08  Interaction: 0.62 | Severe: 1.34 (0.04)  Moderate: 1.44 (0.11)  Mild: 1.34 (0.08) | Severe: 1.34 (0.04)  Moderate: 1.59 (0.10)  Mild: 1.35 (0.07) |
| Discharge to facility or community | Complete  (n=713) | RTP=354  RDP=359 | Group: 0.50  Facility: <0.001  Interaction: 0.45 | Community: 1.41 (0.03)  Facility: 1.06 (0.07) | Community: 1.41 (0.03)  Facility: 1.14 (0.09) |
| Sex | Complete  (n=713) | RTP=354  RDP=359 | Group: 0.66  Sex: 0.66  Interaction: 0.12 | Female: 1.42 (0.06)  Male: 1.32 (0.04) | Female: 1.32 (0.06)  Male: 1.38 (0.04) |
| Race (4 categories) | Complete  (n=712) | RTP=353  RDP=359 | Group: 0.69  Race: 0.42  Interaction: 0.59 | White: 1.35 (0.04)  Black: 1.32 (0.06)  Hispanic: 1.34 (0.12)  Other:1.41 (0.14) | White:1.40 (0.04)  Black: 1.23 (0.08)  Hispanic: 1.39 (0.09)  Other: 1.29 (0.11) |
| Presence of prior limitations | Complete  (n=713) | RTP=354  RDP=359 | Group: 0.72  Limitation: 0.002  Interaction: 0.23 | Yes: 1.30 (0.05)  No: 1.39 (0.04) | Yes: 1.26 (0.05)  No: 1.45 (0.04) |
| Rural vs. Urban/Suburban | Complete  (n=704) | RTP=350  RDP=354 | Group: 0.68  Rural: 0.02  Interaction: 0.71 | Rural: 1.43 (0.05)  Urban/Sub: 1.30 (0.04) | Rural: 1.44 (0.06)  Urban/Sub: 1.34 (0.04) |
| Type of Insurance (Medicare; Medicaid; Private, Other) | Complete  (n=713) | RTP=354  RDP=359 | Group: 0.15  Insurance: <0.001  Interaction: 0.22 | Medicare: 0.99 (0.05)  Medicaid: 1.21 (0.06)  Private: 1.60 (0.04)  Other: 1.35 (0.08) | Medicare:1.10 (0.05)  Medicaid: 1.16 (0.06)  Private: 1.58 (0.05)  Other: 1.56 (0.11) |
| Age | Complete  (n=713) | RTP=354  RDP=359 | Group: 0.66  Age: <0.001  Interaction: 0.75 | Mean (Age= 47.16): 1.34 (0.03)  Decreases w/ increasing age | Mean (Age= 47.16): 1.36 (0.03)  Decreases w/ increasing age |
| FIM Cognitive at discharge | Complete  (n=641) | RTP=312  RDP=329 | Group: >0.99  FIM Cog: <0.001  Interaction: 0.95 | Mean (FIM Cog= 23.12): 1.36 (0.03)  Increases w/ increasing FIM | Mean (FIM Cog= 23.12): 1.37 (0.03)  Increases w/ increasing FIM |
| FIM Motor at discharge | Complete  (n=707) | RTP=351  RDP=356 | Group: 0.36  FIM M: <0.001  Interaction: 0.49 | Mean (FIM M= 64.45): 1.34 (0.03)  Increases w/ increasing FIM | Mean (FIM M= 64.45): 1.37 (0.03)  Increases w/ increasing FIM |
| Having an enrolled caregiver | Complete  (n=713) | RTP=354  RDP=359 | Group: 0.90  Caregiver: 0.51  Interaction: 0.10 | Have caregiver:1.31 (0.04)  No caregiver:1.42 (0.05) | Have caregiver: 1.38 (0.04)  No caregiver: 1.33 (0.05) |
| COVID period | Complete  (n=735) | RTP=354  RDP=359 | Intervention: 0.81  COVID period: 0.29  Interaction: 0.62 | Prior*: 1.36 (0.05)  Prior/After*: 1.32 (0.06)  After*: 1.35 (0.05) | Prior*: 1.38 (0.06)  Prior/After*: 1.28 (0.05)  After*: 1.41 (0.06) |

Abbreviations: RTP, Rehabilitation Transition Plan; RDP, Rehabilitation Discharge Plan; PTA, Post-traumatic Amnesia; FIM, Functional Independence Measure

* Prior= completed study prior to pandemic; Prior/After: Started before and finished during pandemic; After: Started and finished after during pandemic
