# Supplementary material for: Comparing Two Models of Transition from Inpatient Rehabilitation Following Traumatic Brain Injury: A Pragmatic Comparative Effectiveness Trial
Source: J Neurotrauma. Author manuscript; Available in PMC 2026 Jun 25. (PMC13296878; doi:10.1177/08977151251374298)
Supplement: Supplemental Table 5 [file NIHMS2162225-supplement-Supplemental_Table_5.docx]

**Supplemental Table 5. Results of heterogeneity analysis for QOLIBRI at 12-month follow-up**

| **Explanatory Variable** | **Data used** | **Sample Size** | **P-value for** | **Intervention Group Estimated Means (SE)** | |
| --- | --- | --- | --- | --- | --- |
|  |  |  |  | **RTP** | **RDP** |
| Center | Complete  (n=595) | RTP=292  RDP=303 | Group: 0.32  Center: 0.009  Interaction: 0.12 | Site A: 67.1 (2.5)  Site B: 71.3 (2.5)  Site C: 66.9 (2.7)  Site D: 59.1 (2.3)  Site E: 64.7 (4.4)  Site F: 67.3 (2.7) | Site A: 75.1 (2.4)  Site B: 66.2 (2.4)  Site C: 69.3 (3.2)  Site D: 66.1 (2.6)  Site E: 61.4 (4.4)  Site F: 68.5 (2.5) |
| PTA severity | Complete  (n=538) | RTP=262  RDP=276 | Group: 0.09  Severity: 0.10  Interaction: 0.28 | Severe: 66.0 (1.5)  Moderate: 57.7 (3.7)  Mild: 68.7 (2.4) | Severe: 66.8 (1.6)  Moderate: 67.1 (3.7)  Mild: 69.6 (2.3) |
| Discharge to facility or community (Disposition) | Complete  (n=595) | RTP=292  RDP=303 | Group: 0.99  Facility: 0.12  Interaction: 0.11 | Community: 65.7 (1.2)  Facility: 65.9 (2.7) | Community: 69.3 (1.2)  Facility: 62.2 (3.4) |
| Sex | Complete  (n=595) | RTP=292  RDP=303 | Group: 0.21  Sex: 0.04  Interaction: 0.55 | Female: 63.8 (2.2)  Male: 66.4 (1.3) | Female: 65.0 (2.3)  Male: 69.8 (1.3) |
| Race (4 categories) | Complete  (n=595) | RTP=292  RDP=303 | Group: 0.03  Race: 0.78  Interaction: 0.40 | White: 66.0 (1.3)  Black: 68.1 (2.7)  Hispanic: 60.7 (5.7)  Other: 62.6 (4.9) | White: 67.5 (1.4)  Black: 69.2 (3.0)  Hispanic: 70.2 (3.3)  Other: 71.6 (5.0) |
| Presence of prior limitations | Complete  (n=595) | RTP=292  RDP=303 | Group: 0.08  Limitation: <0.001  Interaction: 0.28 | Yes: 60.9 (1.7)  No: 69.6 (1.4) | Yes: 65.5 (1.8)  No: 70.7 (1.5) |
| Rural vs. Urban/Suburban | Complete  (n=588) | RTP=289  RDP=299 | Group: 0.06  Rural: 0.15  Interaction: 0.23 | Rural: 66.0 (1.80)  Urban/Sub: 65.6 (1.5) | Rural: 71.4 (2.1)  Urban/Sub: 66.8 (1.4) |
| Type of Insurance (Medicare; Medicaid; Private, Other) | Complete  (n=595) | RTP=292  RDP=303 | Group: 0.08  Insurance: <0.001  Interaction: 0.88 | Medicare: 64.5 (2.5)  Medicaid: 59.7 (2.6)  Private:69.4 (1.5)  Other: 66.1 (3.5) | Medicare: 66.7 (2.4)  Medicaid: 62.8 (2.8)  Private: 71.1 (1.6)  Other: 71.8 (3.1) |
| Age | Complete  (n=595) | RTP=292  RDP=303 | Group: 0.75  Age: 0.08  Interaction: 0.77 | Mean (Age= 47.04): 65.7 (1.2)  Decreases w/ increasing age | Mean (Age= 47.04): 68.3 (1.1)  Decreases w/ increasing age |
| FIM Cognitive at discharge | Complete  (n=529) | RTP=252  RDP=277 | Intervention: 0.76  FIM Cog: 0.05  Interaction: 0.58 | Mean (FIM Cog= 24.57): 66.3 (1.3)  Increases with increasing FIM | Mean (FIM Cog= 24.57): 68.3 (1.2)  Increases with increasing FIM |
| FIM Motor at discharge | Complete  (n=591) | RTP=289  RDP=302 | Intervention: 0.49  FIM M: <0.001  Interaction: 0.76 | Mean (FIM M= 67.72): 65.6 (1.2)  Increases with increasing FIM | Mean (FIM M= 67.72): 68.4 (1.1)  Increases with increasing FIM |
| Having an enrolled caregiver | Complete  (n=595) | RTP= 292  RDP=303 | Group: 0.16  Caregiver: 0.86  Interaction: 0.52 | Have caregiver: 65.2 (1.5)  No caregiver: 66.6 (1.9) | Have caregiver: 68.7 (1.5)  No caregiver: 67.9 (1.8) |
| COVID period | Complete  (n=595) | RTP=292  RDP=303 | Intervention: 0.14  COVID period: 0.44  Interaction: 0.74 | Prior*: 64.7 (2.4)  Prior/After*: 67.1 (2.2)  After*: 65.6 (1.5) | Prior*: 66.3 (2.3)  Prior/After*: 68.6 (2.0)  After*: 69.8 (1.8) |

Abbreviations: RTP, Rehabilitation Transition Plan; RDP, Rehabilitation Discharge Plan; PTA, Post-traumatic Amnesia; FIM, Functional Independence Measure

* Prior= completed study prior to pandemic; Prior/After: Started before and finished during pandemic; After: Started and finished after during pandemic
