# Supplementary material for: Comparing Two Models of Transition from Inpatient Rehabilitation Following Traumatic Brain Injury: A Pragmatic Comparative Effectiveness Trial
Source: J Neurotrauma. Author manuscript; Available in PMC 2026 Jun 25. (PMC13296878; doi:10.1177/08977151251374298)
Supplement: Supplemental Appendix [file NIHMS2162225-supplement-Supplemental_Appendix.docx]

#### Appendix A: COVID-19 Analyses

#### Primary Outcome: PART-O-17

We performed an ANOVA for each follow-up time, with intervention and study period as the explanatory variables, including an interaction of both variables. Table A1 below shows the mean (SD) of PART-O-17 at each follow-up by intervention and study period. The last column contains the p-values for the ANOVA and comparisons of pairs of the means of periods (using Bonferroni method).

A linear mixed-effects model was also used to assess the longitudinal impact the COVID-19 impact may have had. This model included fixed effects for treatment assignment, time of measurement, COVID classification, and all two- and three-way interactions between these variables. A significant three-way interaction would indicate that difference in trajectories between treatment groups was impacted by COVID-19 classification.

Figure A1 shows the estimated trajectories for PART-O-17 stratified by treatment assignment and COVID-19 classification.

Figure A1. PART-O-17 scores by treatment and COVID-19 classification


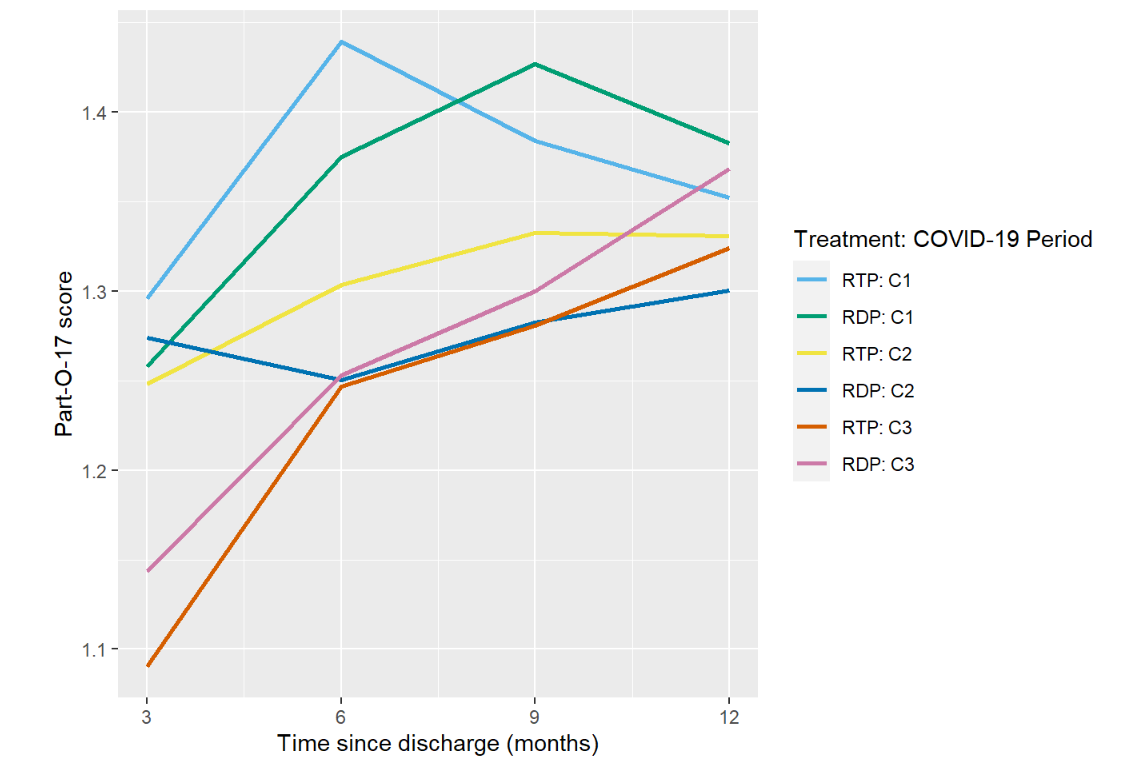


Table A1. PART-O-17 scores by treatment and COVID-19 classification

| Follow-up | Intervention | COVID Period | N | PART-O-17 Mean (SD) | ANOVA p-values and comparison of means for periods |
| --- | --- | --- | --- | --- | --- |
| 3 Months | RTP | 1 | 113 | 1.29 (.53) | Intervention: p= 0.79  COVID Period: p<0.01  Interaction: p=0.52  Periods:  1 vs. 2: p>0.99  1 vs. 3: p=0.005  2 vs. 3: p=0.004 |
|  |  | 2 | 97 | 1.28 (.54) |  |
|  |  | 3 | 172 | 1.09 (.49) |  |
|  | RDP | 1 | 115 | 1.25 (.55) |  |
|  |  | 2 | 106 | 1.28 (.56) |  |
|  |  | 3 | 163 | 1.16 (.56) |  |
| 6 Months | RTP | 1 | 109 | 1.46 (.53) | Intervention: p=0.39  COVID Period: p=0.006  Interaction: p=0.70  Periods:  1 vs. 2: p=0.03  1 vs. 3: p=0.01  2 vs. 3: p>0.99 |
|  |  | 2 | 99 | 1.27 (.60) |  |
|  |  | 3 | 164 | 1.27 (.57) |  |
|  | RDP | 1 | 110 | 1.37 (.57) |  |
|  |  | 2 | 98 | 1.26 (.60) |  |
|  |  | 3 | 155 | 1.26 (.63) |  |
| 9 Months | RTP | 1 | 109 | 1.39 (.56) | Intervention: p=0.94  COVID Period: p=0.14  Interaction: p=0.54  Periods:  1 vs. 2: p=0.25  1 vs. 3: p=0.21  2 vs. 3: p>0.99 |
|  |  | 2 | 85 | 1.35 (.62) |  |
|  |  | 3 | 163 | 1.30 (.57) |  |
|  | RDP | 1 | 110 | 1.44 (.59) |  |
|  |  | 2 | 103 | 1.28 (.57) |  |
|  |  | 3 | 150 | 1.33 (.63) |  |
| 12 Months | RTP | 1 | 104 | 1.36 (.55) | Intervention: p=0.81  COVID Period: p=0.29  Interaction: p=0.62  Periods:  1 vs. 2: p=0.69  1 vs. 3: p>0.99  2 vs. 3: p=0.38 |
|  |  | 2 | 96 | 1.32 (.62) |  |
|  |  | 3 | 154 | 1.35 (.56) |  |
|  | RDP | 1 | 106 | 1.38 (.58) |  |
|  |  | 2 | 104 | 1.28 (.54) |  |
|  |  | 3 | 149 | 1.41 (.68) |  |

Abbreviations: RTP, Rehabilitation Transition Plan; RDP, Rehabilitation Discharge Plan

If we look at each follow-up separately, there were no interactions between the interventions and the pandemic period in which the individual was in the study. The COVID period was statistically significant at 3- and 6-month follow-ups. At 3-month follow-up, individuals who started in the pre-COVID period (groups 1 and 2), regardless of intervention, reported higher participation at 3 months than individuals who started during the pandemic. At 6-month follow-up, individuals who started and finished in the pre-COVID period (group 1), regardless of intervention, reported higher participation at 6 months than individuals who had some part of the study during the pandemic (groups 2 and 3). At 9 and 12 months, the three groups did not differ statistically in mean participation. Furthermore, there was not significant evidence that COVID-19 impacted the difference in trajectories between treatment groups (complete linear mixed effect model results in Table A2.)

Table A2. Results from a linear mixed effects model analyzing the impact of COVID-19 on PART-O-17 scores

|  | Estimate | Std. Error | Pr(>\|t\|) |
| --- | --- | --- | --- |
| Intercept | 1.296 | 0.050 | 0.000 |
| Treatment (reference: RTP) | | | |
| RDP | -0.038 | 0.071 | 0.592 |
| Time (reference: 3 months) | | | |
| Month 6 | 0.143 | 0.037 | 0.000 |
| Month 9 | 0.088 | 0.041 | 0.030 |
| Month 12 | 0.056 | 0.046 | 0.226 |
| COVID Period (reference: COVID 1 [completed study pre-covid]) | | | |
| COVID 2 | -0.048 | 0.073 | 0.514 |
| COVID 3 | -0.206 | 0.065 | 0.002 |
| Treatment:Time interaction | | | |
| RDP:Month 6 | -0.026 | 0.053 | 0.618 |
| RDP:Month 9 | 0.081 | 0.057 | 0.160 |
| RDP:Month 12 | 0.069 | 0.065 | 0.291 |
| Treatment:COVID period interaction | | | |
| RDP:COVID 2 | 0.064 | 0.103 | 0.533 |
| RDP:COVID 3 | 0.092 | 0.093 | 0.323 |
| Time:COVID period interaction | | | |
| Month 6:COVID 2 | -0.088 | 0.055 | 0.108 |
| Month 9:COVID 2 | -0.004 | 0.060 | 0.949 |
| Month 12:COVID 2 | 0.027 | 0.067 | 0.691 |
| Month 6:COVID 3 | 0.013 | 0.048 | 0.783 |
| Month 9:COVID 3 | 0.102 | 0.052 | 0.051 |
| Month 12:COVID 3 | 0.178 | 0.060 | 0.003 |
| Treatment:Time:COVID period interaction (p-value = 0.616) | | | |
| RDP:Month 6:COVID 2 | -0.053 | 0.077 | 0.494 |
| RDP:Month 9:COVID 2 | -0.156 | 0.084 | 0.063 |
| RDP:Month 12:COVID 2 | -0.125 | 0.094 | 0.184 |
| RDP:Month 6:COVID 3 | -0.021 | 0.068 | 0.761 |
| RDP:Month 9:COVID 3 | -0.115 | 0.074 | 0.123 |
| RDP:Month 12:COVID 3 | -0.078 | 0.085 | 0.358 |

#### Primary Outcome: QOLIBRI (using the percentage score)

We performed an ANOVA for each follow-up time and intervention and COVID period as the explanatory variables, including an interaction of both variables. Table A3 below shows the mean (SD) of QOLIBRI at each follow-up by intervention and COVID period. The last column contains the p-values for the ANOVA and comparisons of pairs of the means of periods (using Bonferroni method).

A linear mixed-effects model was used to assess the longitudinal impact the COVID-19 impact may have had (Figure A3). This model included fixed effects for treatment assignment, time of measurement, COVID classification, and all two- and three-way interactions between variables. A significant three-way interaction would indicate that difference in trajectories between treatment groups was impacted by COVID-19 classification.

Figure A3: QOLIBRI scores by treatment and COVID-19 classification.


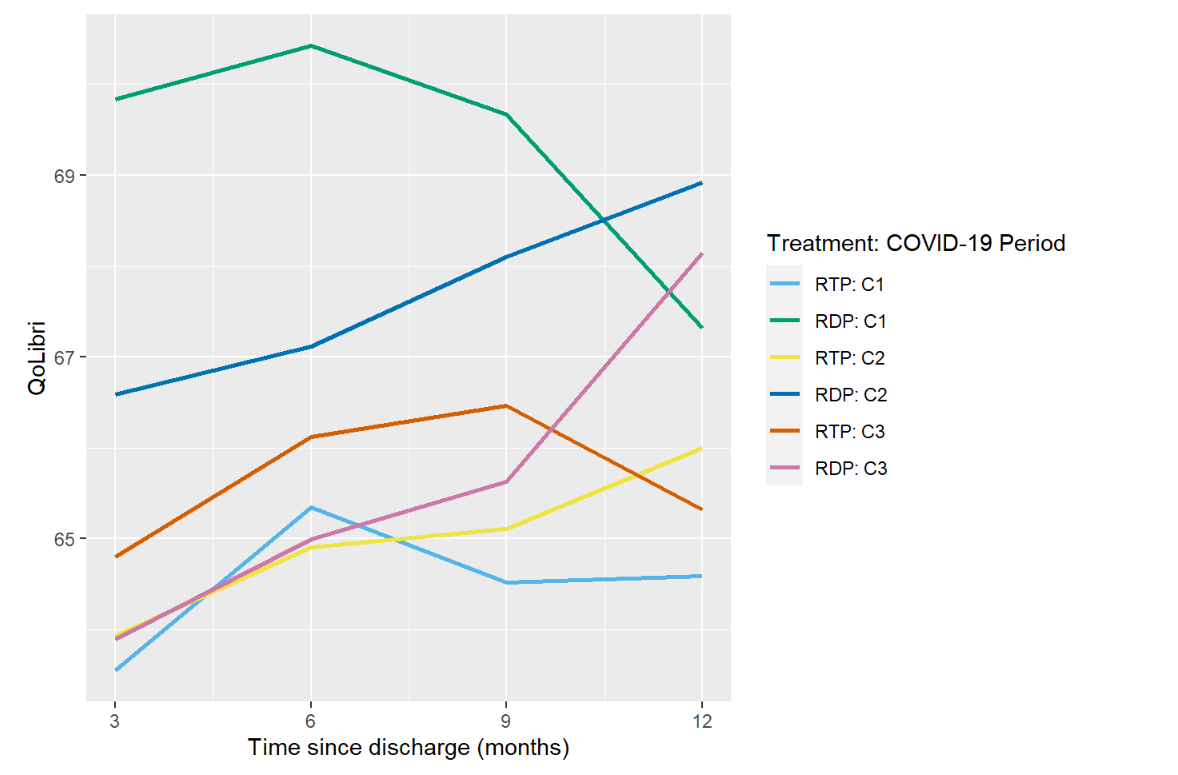


Table A3. QOLIBRI scores by treatment and COVID-19 classification

| Follow-up | Intervention | COVID Period | N | QOLIBRI Mean (SD) | ANOVA p-values and comparison of means for periods |
| --- | --- | --- | --- | --- | --- |
| 3 Months | RTP | 1 | 92 | 65.0 (18.2) | Intervention: p= 0.08  COVID Period: p=0.24  Interaction: p=0.17  Periods:  1 vs. 2: p=0.85  1 vs. 3: p=0.27  2 vs. 3: p>0.99 |
|  |  | 2 | 70 | 64.7 (19.7) |  |
|  |  | 3 | 141 | 65.4 (17.4) |  |
|  | RDP | 1 | 96 | 71.1 (16.5) |  |
|  |  | 2 | 76 | 67.1 (17.5) |  |
|  |  | 3 | 128 | 65.0 (19.2) |  |
| 6 Months | RTP | 1 | 96 | 65.1 (18.7) | Intervention: p=0.07  COVID Period: p=0.55  Interaction: p=0.17  Periods:  1 vs. 2: p>0.99  1 vs. 3: p=0.85  2 vs. 3: p>0.99 |
|  |  | 2 | 77 | 65.5 (17.6) |  |
|  |  | 3 | 142 | 66 .5 (17.4) |  |
|  | RDP | 1 | 92 | 71.4 (19.4) |  |
|  |  | 2 | 80 | 68.2 (17.3) |  |
|  |  | 3 | 119 | 66.0 (20.3) |  |
| 9 Months | RTP | 1 | 90 | 64.6 (19.7) | Intervention: p=0.11  COVID Period: p=0.93  Interaction: p=0.32  Periods:  1 vs. 2: p>0.99  1 vs. 3: p>0.99  2 vs. 3: p>0.99 |
|  |  | 2 | 71 | 66.1 (19.8) |  |
|  |  | 3 | 139 | 66.8 (18.1) |  |
|  | RDP | 1 | 96 | 70.3 (19.5) |  |
|  |  | 2 | 86 | 68.4 (19.6) |  |
|  |  | 3 | 121 | 66.8 (20.5) |  |
| 12 Months | RTP | 1 | 81 | 64.7 (21.7) | Intervention: p=0.14  COVID Period: p=0.44  Interaction: p=0.73  Periods:  1 vs. 2: p=0.84  1 vs. 3: p= 0.87  2 vs. 3: p>0.99 |
|  |  | 2 | 79 | 67.1 (19.7) |  |
|  |  | 3 | 132 | 65.6 (17.6) |  |
|  | RDP | 1 | 96 | 66.3 (22.3) |  |
|  |  | 2 | 90 | 68.6 (19.1) |  |
|  |  | 3 | 117 | 69.8 (19.3) |  |

Abbreviations: RTP, Rehabilitation Transition Plan; RDP, Rehabilitation Discharge Plan

For quality of life, neither intervention, COVID period or their interaction was statistically significant at any follow-up time. However, from the linear mixed effects model, we saw that among the subgroup who completed the study pre-COVID-19, those in the RDP group had significantly higher scores at 3 months than those in the RTP group. There was not significant evidence that COVID-19 impacted the difference in trajectories between treatment groups (complete linear mixed effect model results in Table A4.)

Table A4. Results from a linear mixed effects model analyzing the impact of COVID-19 on QOLIBRI scores

|  | Estimate | Std. Error | Pr(>\|t\|) |
| --- | --- | --- | --- |
| Intercept | 63.546 | 1.851 | 0.000 |
| Treatment (reference: RTP) | | | |
| RDP | 6.287 | 2.607 | 0.016 |
| Time (reference: 3 months) | | | |
| Month 6 | 1.796 | 1.355 | 0.185 |
| Month 9 | 0.969 | 1.519 | 0.523 |
| Month 12 | 1.047 | 1.807 | 0.563 |
| COVID Period (reference: COVID 1 [completed study pre-covid]) | | | |
| COVID 2 | 0.378 | 2.766 | 0.891 |
| COVID 3 | 1.253 | 2.393 | 0.601 |
| Treatment:Time interaction | | | |
| RDP:Month 6 | -1.204 | 1.900 | 0.527 |
| RDP:Month 9 | -1.129 | 2.126 | 0.595 |
| RDP:Month 12 | -3.565 | 2.490 | 0.153 |
| Treatment:COVID period interaction | | | |
| RDP:COVID 2 | -3.626 | 3.847 | 0.346 |
| RDP:COVID 3 | -7.193 | 3.421 | 0.036 |
| Time:COVID period interaction | | | |
| Month 6:COVID 2 | -0.810 | 2.051 | 0.693 |
| Month 9:COVID 2 | 0.215 | 2.307 | 0.926 |
| Month 12:COVID 2 | 1.026 | 2.659 | 0.700 |
| Month 6:COVID 3 | -0.476 | 1.740 | 0.785 |
| Month 9:COVID 3 | 0.699 | 1.956 | 0.721 |
| Month 12:COVID 3 | -0.530 | 2.309 | 0.819 |
| Treatment:Time:COVID period interaction (p-value = 0.265) | | | |
| RDP:Month 6:COVID 2 | 0.749 | 2.872 | 0.794 |
| RDP:Month 9:COVID 2 | 1.466 | 3.208 | 0.648 |
| RDP:Month 12:COVID 2 | 3.825 | 3.681 | 0.299 |
| RDP:Month 6:COVID 3 | 0.982 | 2.483 | 0.693 |
| RDP:Month 9:COVID 3 | 1.195 | 2.778 | 0.667 |
| RDP:Month 12:COVID 3 | 7.304 | 3.251 | 0.025 |

Abbreviations: RTP, Rehabilitation Transition Plan; RDP, Rehabilitation Discharge Plan

From the two outcomes, it seems that participation score at 3- and 6-month follow-ups were higher in the groups that started before the pandemic, especially for group 1 at 3 and 6 months, and group 2 at 3 months.
