# Supplementary material for: Comparing Two Models of Transition from Inpatient Rehabilitation Following Traumatic Brain Injury: A Pragmatic Comparative Effectiveness Trial
Source: J Neurotrauma. Author manuscript; Available in PMC 2026 Jun 25. (PMC13296878; doi:10.1177/08977151251374298)
Supplement: Supplemental Table 3 [file NIHMS2162225-supplement-Supplemental_Table_3.docx]

**Supplemental Table 3. Results of heterogeneity analysis for QOLIBRI at 6-month follow-up**

| **Explanatory Variable** | **Data used** | **Sample Size** | **P-value for** | **Intervention Group Estimated Means (SE)** | |
| --- | --- | --- | --- | --- | --- |
|  |  |  |  | **RTP** | **RDP** |
| Center | Complete  (n=606) | RTP=315  RDP=291 | Group: 0.12  Center: 0.05  Interaction: 0.16 | Site A: 66.1 (2.2)  Site B: 70.3 (2.4)  Site C: 66.1 (2.4)  Site D: 64.1 (2.0)  Site E: 60.0 (3.7)  Site F: 66.9 (2.4) | Site A: 75.2 (2.1)  Site B: 65.6 (2.6)  Site C: 70.2 (2.9)  Site D: 67.7 (2.6)  Site E: 62.8 (3.6)  Site F: 66.4 (2.6) |
| PTA severity | Complete  (n=551) | RTP=286  RDP=265 | Group: 0.38  Severity: 0.46  Interaction: 0.60 | Severe: 65.9 (1.2)  Moderate: 63.0 (3.3)  Mild: 68.0 (2.4) | Severe: 68.9 (1.4)  Moderate: 66.1 (3.5)  Mild: 67.1 (2.6) |
| Discharge to facility or community (Disposition) | Complete  (n=606) | RTP=315  RDP=291 | Group: 0.50  Facility: 0.22  Interaction: 0.59 | Community: 66.1 (1.1)  Facility: 64.5 (2.7) | Community: 68.8 (1.2)  Facility: 64.8 (3.7) |
| Sex | Complete  (n=606) | RTP=315  RDP=291 | Group: 0.04  Sex: 0.001  Interaction: 0.27 | Female: 60.4 (2.0)  Male: 67.7 (1.1) | Female: 65.8 (2.1)  Male: 69.4 (1.3) |
| Race (4 categories) | Complete  (n=605) | RTP=15  RDP=290 | Group: 0.05  Race: 0.40  Interaction: 0.50 | White: 66.1 (1.2)  Black: 68.3 (2.6)  Hispanic: 60.2 (4.0)  Other: 62.6 (4.5) | White: 68.6 (1.3)  Black: 67.8 (3.1)  Hispanic: 65.8 (3.2)  Other: 72.3 (5.4) |
| Presence of prior limitations | Complete  (n=605) | RTP=315  RDP=290 | Group: 0.07  Limitation: <0.001  Interaction: 0.49 | Yes: 62.9 (1.5)  No: 68.2 (1.3) | Yes: 64.5 (1.6)  No: 71.9 (1.5) |
| Rural vs. Urban/Suburban | Complete  (n=599) | RTP=312  RDP=287 | Group: 0.06  Rural: 0.10  Interaction: 0.17 | Rural: 66.3 (1.6)  Urban/Sub: 65.8 (1.3) | Rural: 71.5 (2.0)  Urban/Sub: 66.6 (1.4) |
| Type of Insurance (Medicare; Medicaid; Private, Other) | Complete  (n=606) | RTP=315  RDP=291 | Group: 0.08  Insurance: 0.009  Interaction: 0.52 | Medicare: 66.5 (2.5)  Medicaid: 62.9 (2.2)  Private: 66.9 (1.3)  Other: 66.9 (2.8) | Medicare: 68.8 (2.0)  Medicaid: 62.2 (2.9)  Private: 69.3 (1.7)  Other: 74.5 (3.1) |
| Age | Complete  (n=606) | RTP=315  RDP=291 | Group: 0.55  Age: 0.003  Interaction: 0.95 | Mean (Age= 47.16): 65.8 (1.0)  Decreases w/ increasing age | Mean (Age= 47.16): 68.4 (1.1)  Decreases w/ increasing age |
| FIM Cognitive at discharge | Complete  (n=542) | RTP=275  RDP=267 | Group: 0.32  FIM Cog: 0.03  Interaction:0.48 | Mean (FIM Cog= 24.70): 66.0 (1.1)  Increases with increasing FIM | Mean (FIM Cog= 24.70): 68.3 (1.1)  Increases with increasing FIM |
| FIM Motor at discharge | Complete  (n=602) | RTP=312  RDP=290 | Group: 0.12  FIM M: 0.003  Interaction: 0.22 | Mean (FIM M= 68.34): 65.8 (1.0)  Increases with increasing FIM | Mean (FIM M= 68.34): 68.3 (1.1)  Increases with increasing FIM |
| Having an enrolled caregiver | Complete  (n=606) | RTP=315  RDP=291 | Group: 0.30  Caregiver: 0.60  Interaction: 0.02 | Have caregiver: 64.2 (1.2)  No caregiver: 68.7 (1.5) | Have caregiver: 69.5 (1.4)  No caregiver: 66.6 (1.69) |
| COVID Period | Complete  (n=606) | RTP=315  RDP=291 | Intervention: 0.07  COVID period: 0.53  Interaction: 0.17 | Prior*: 65.1 (1.9)  Prior/After*: 65.6 (2.0)  After*: 66.5 (1.5) | Prior*: 71.4 (2.0)  Prior/After*: 68.2 (1.9)  After*: 66.0 (1.9) |

Abbreviations: RTP, Rehabilitation Transition Plan; RDP, Rehabilitation Discharge Plan; PTA, Post-traumatic Amnesia; FIM, Functional Independence Measure

* Prior= completed study prior to pandemic; Prior/After: Started before and finished during pandemic; After: Started and finished after during pandemic
