# Supplementary material for: Comparing Two Models of Transition from Inpatient Rehabilitation Following Traumatic Brain Injury: A Pragmatic Comparative Effectiveness Trial
Source: J Neurotrauma. Author manuscript; Available in PMC 2026 Jun 25. (PMC13296878; doi:10.1177/08977151251374298)
Supplement: Supplemental Table 2 [file NIHMS2162225-supplement-Supplemental_Table_2.docx]

**Supplemental Table 2. Results of heterogeneity analysis for PART-O-17 at 6-month follow-up**

| **Explanatory Variable** | **Data used** | **Sample Size** | **P-value for** | **Intervention Group Estimated Means (SE)** | |
| --- | --- | --- | --- | --- | --- |
|  |  |  |  | **RTP** | **RDP** |
| Center | Complete  (n=735) | RTP=372  RDP=363 | Group: 0.44  Center: <0.001  Interaction: 0.21 | Site A: 1.34 (0.06)  Site B: 1.47 (0.07)  Site C: 1.39 (0.08)  Site D: 1.39 (0.07)  Site E: 1.05 (0.09)  Site F: 1.22 (0.07) | Site A: 1.46 (0.07)  Site B: 1.31 (0.08)  Site C: 1.29 (0.08)  Site D: 1.50 (0.06)  Site E: 1.00 (0.08)  Site F: 1.11 (0.07) |
| PTA severity | Complete  (n=671) | RTP=340  RDP=331 | Group: 0.86  Severity: 0.16  Interaction: 0.28 | Severe: 1.32 (0.04)  Moderate: 1.30 (0.09)  Mild: 1.42 (0.08) | Severe: 1.25 (0.04)  Moderate: 1.47 (0.10)  Mild: 1.35 (0.07) |
| Discharge to facility or community | Complete  (n=735) | RTP=372  RDP=363 | Group: 0.36  Facility: <0.001  Interaction: 0.59 | Community: 1.39 (0.03)  Facility: 1.03 (0.08) | Community: 1.37 (0.03)  Facility: 0.94 (0.07) |
| Sex | Complete  (n=735) | RTP=372  RDP=363 | Group: 0.30  Sex: 0.23  Interaction: 0.35 | Female: 1.32 (0.06)  Male: 1.33 (0.03) | Female: 1.22 (0.05)  Male: 1.33 (0.04) |
| Race (4 categories) | Complete  (n=733) | RTP=371  RDP=362 | Group: 0.59  Race: 0.03  Interaction: 0.93 | White: 1.36 (0.04)  Black: 1.23 (0.07)  Hispanic: 1.28 (0.11)  Other:1.34 (0.14) | White:1.34 (0.04)  Black: 1.14 (0.08)  Hispanic: 1.28 (0.08)  Other: 1.31 (0.12) |
| Presence of prior limitations | Complete  (n=734) | RTP=372  RDP=362 | Group: 0.57  Limitation: <0.001  Interaction: 0.86 | Yes: 1.23 (0.04)  No: 1.41 (0.04) | Yes: 1.21 (0.05)  No: 1.38 (0.04) |
| Rural vs. Urban/Suburban | Complete  (n=727) | RTP=369  RDP=358 | Group: 0.35  Rural: 0.08  Interaction: 0.30 | Rural: 1.42 (0.05)  Urban/Sub: 1.28 (0.04) | Rural: 1.32 (0.06)  Urban/Sub: 1.29 (0.04) |
| Type of Insurance (Medicare; Medicaid; Private, Other) | Complete  (n=735) | RTP=372  RDP=363 | Group: 0.98  Insurance: <0.001  Interaction: 0.81 | Medicare:1.11 (0.06)  Medicaid: 1.20 (0.06)  Private: 1.50 (0.04)  Other: 1.30 (0.07) | Medicare:1.11 (0.05)  Medicaid: 1.16 (0.06)  Private: 1.46 (0.05)  Other: 1.39 (0.10) |
| Age | Complete  (n=735) | RTP=372  RDP=363 | Group: 0.70  Age: <0.001  Interaction: 0.50 | Mean (Age= 47.12): 1.33 (0.03)  Decreases w/ increasing age | Mean (Age= 47.12): 1.30 (0.03)  Decreases w/ increasing age |
| FIM Cognitive at discharge | Complete  (n=655) | RTP=331  RDP=334 | Group: 0.15  FIM Cog: <0.001  Interaction: 0.22 | Mean (FIM Cog=23.15): 1.34 (0.03)  Increases w/ increasing FIM | Mean (FIM Cog=23.15): 1.30 (0.03)  Increases w/ increasing FIM |
| FIM Motor at discharge | Complete  (n=729) | RTP=369  RDP=360 | Group: 0.59  FIM M: <0.001  Interaction: 0.72 | Mean (FIM Mot=64.53): 1.33 (0.03)  Increases w/ increasing FIM | Mean (FIM Mot=64.53): 1.30 (0.03)  Increases w/ increasing FIM |
| Having an enrolled caregiver | Complete  (n=735) | RTP=372  RDP=363 | Group: 0.15  Caregiver: 0.002  Interaction: 0.04 | Have caregiver: 1.25 (0.05)  No caregiver: 1.49 (0.04) | Have caregiver: 1.28 (0.04)  No caregiver: 1.33 (0.05) |
| COVID period | Complete  (n=735) | RTP=372  RDP=363 | Intervention: 0.39  COVID period: 0.006  Interaction: 0.70 | Prior*: 1.46 (0.05)  Prior/After*: 1.27 (0.06)  After*: 1.27 (0.04) | Prior*: 1.37 (0.05)  Prior/After*: 1.26 (0.06)  After*: 1.26 (0.05) |

Abbreviations: RTP, Rehabilitation Transition Plan; RDP, Rehabilitation Discharge Plan; PTA, Post-traumatic Amnesia; FIM, Functional Independence Measure

* Prior= completed study prior to pandemic; Prior/After: Started before and finished during pandemic; After: Started and finished after during pandemic
