# Supplementary material for: Comparing Two Models of Transition from Inpatient Rehabilitation Following Traumatic Brain Injury: A Pragmatic Comparative Effectiveness Trial
Source: J Neurotrauma. Author manuscript; Available in PMC 2026 Jun 25. (PMC13296878; doi:10.1177/08977151251374298)
Supplement: Supplemental Table 1 [file NIHMS2162225-supplement-Supplemental_Table_1.docx]

Supplemental Table 1. Differences between individuals who completed PART-O-17 at 6-month follow-up and individuals with missing data (percentages add to 100% by row)

| **Characteristic** | **Not Missing** | **Missing due to other reasons** | **Missing due death prior 6M** | **P-value** |
| --- | --- | --- | --- | --- |
| Sample size | 735 | 166 | 24 |  |
| Age, mean (SD) | 47 (20) | 47 (20) | 58 (20) | 0.03 |
| Sex, n (%) |  |  |  |  |
| Females | 196 (79.0) | 44 (17.7) | 8 (3.2) | 0.74 |
| Males | 539 (79.6) | 122 (18.0) | 16 (2.4) |  |
| Education level, n (%) |  |  |  |  |
| Less than HS | 90 (72.0) | 30 (24.0) | 5 (4.0) | 0.23 |
| HS Diploma | 259 (82.7) | 48 (15.3) | 6 (1.9) |  |
| Some College | 195(79.9) | 44 (18.0) | 5 (2.0) |  |
| College | 189 (82.5) | 34 (14.8) | 6 (2.6) |  |
| Married/Partner, n (%) |  |  |  |  |
| Married/significant other | 296 (85.1) | 44 (12.6) | 8 (2.3) | 0.01 |
| Single | 438 (77.1) | 115 (20.2) | 15 (2.6) |  |
| Hispanic, n (%) |  |  |  |  |
| No | 660 (80.0) | 145 (17.6) | 20 (2.4) | 0.39 |
| Yes | 57 (75.0) | 18 (23.7) | 1 (1.3) |  |
| Race, n (%) |  |  |  |  |
| White | 496 (81.3) | 100 (16.4) | 14 (2.3) | 0.47 |
| Black | 127 (79.4) | 30 (18.8) | 3 (1.9) |  |
| Hispanic or other | 110 (75.9) | 29 (20.0) | 6 (4.1) |  |
| Competitively employed, n (%) |  |  |  |  |
| Yes | 450 (81.5) | 91 (16.5) | 11 (2.0) | 0.26 |
| No | 285 (77.7) | 70 (19.1) | 12 (3.3) |  |
| Earnings, n (%) |  |  |  |  |
| None | 249 (77.3) | 62 (19.3) | 11 (3.4) | 0.48 |
| Less than 30K | 135 (80.4) | 28 (16.7) | 5 (3.0) |  |
| 30K to less than 60K | 150 (85.2) | 24 (13.6) | 2 (1.1) |  |
| 60K or more | 131 (78.9) | 31 (18.7) | 4 (2.4) |  |
| English language, n (%) |  |  |  |  |
| English | 688 (79.9) | 151 (17.5) | 22 (2.6) | 0.81 |
| Spanish | 24 (82.8) | 5 (17.2) | 0 (0.0) |  |
| Other | 23 (85.2) | 3 (11.1) | 1 (3.7) |  |
| Prior limitation, n (%) |  |  |  |  |
| No | 388 (80.2) | 90 (18.6) | 6 (1.2) | 0.02 |
| Yes | 346 (80.3) | 68 (15.8) | 17 (3.9) |  |
| Cause, n (%) |  |  |  |  |
| Vehicle (plus pedestrian/bike) | 392 (81.7) | 80 (16.7) | 8 (1.7) | 0.05 |
| Fall | 252 (80.3) | 52 (16.6) | 10 (3.2) |  |
| Violence | 54 (66.7) | 24 (29.6) | 3 (3.7) |  |
| Other causes | 32 (84.2) | 5 (13.2) | 1 (2.6) |  |
| GCS total severity, n (%) |  |  |  |  |
| Severe | 94 (78.3) | 22 (18.3) | 4 (3.3) | 0.08 |
| Moderate | 57 (68.7) | 22 (26.5) | 4 (4.8) |  |
| Mild | 227 (79.4) | 51 (17.8) | 8 (2.8) |  |
| Sedated/Intubated | 295 (83.3) | 54 (15.3) | 5 (1.4) |  |
| SCI, n (%) |  |  |  |  |
| No | 637 (79.6) | 143 (17.9) | 20 (2.5) | 0.70 |
| Yes | 97 (83.6) | 17 (14.7) | 2 (1.7) |  |
| Discharged to a facility, n (%) |  |  |  |  |
| Yes (facility) | 124 (69.7) | 44 (24.7) | 10 (5.6) | <0.001 |
| No (Home/community) | 611 (81.8) | 122 (16.3) | 14 (1.9) |  |
| Insurance Type, n (%) |  |  |  |  |
| Medicare | 170 (78.7) | 36 (16.7) | 10 (4.6) | 0.07 |
| Medicaid | 171 (75.7) | 49 (21.7) | 6 (2.7) |  |
| Private/Self | 310 (81.8) | 64 (16.9) | 5 (1.3) |  |
| Other (Workers Comp., others) | 84 (86.6) | 12 (12.4) | 1 (1.0) |  |
| Rehab length of stay, mean (SD) | 25.7 (24.3) | 25.7 (19.5) | 38.1 (40.0) | 0.06 |
| FIM Cognitive at Discharge, mean (SD) | 23.2 (6.6) | 22.6 (6.8) | 18.5 (8.7) | 0.02 |
| FIM Motor at Discharge, mean (SD) | 64.5 (19.0) | 61.8 (22.3) | 50.0 (23.3) | 0.002 |
| Caregiver enrolled?, n (%) |  |  |  |  |
| No | 251 (73.8) | 80 (23.5) | 9 (2.6) | 0.003 |
| Yes | 484 (82.7) | 86 (14.7) | 15 (2.6) |  |
| COVID period |  |  |  |  |
| Started and finished study prior to pandemic (3/1/2020) | 219 (81.1) | 49 (18.1) | 2 (0.7) | 0.003 |
| Started pre-pandemic and finished during pandemic | 197 (74.3) | 62 (23.4) | 6 (2.3) |  |
| Started and finished study during pandemic | 319 (81.8) | 55 (14.1) | 16 (4.1) |  |

Abbreviations: GCS, Glasgow Coma Scale; SCI, spinal cord injury; FIM, Functional Independence Measure
